# Exploiting the Multifunctionality of M^2+^/Imidazole–Etidronates for Proton Conductivity (Zn^2+^) and Electrocatalysis (Co^2+^, Ni^2+^) toward the HER, OER, and ORR

**DOI:** 10.1021/acsami.1c21876

**Published:** 2022-02-22

**Authors:** Álvaro Vílchez-Cózar, Eirini Armakola, Maria Gjika, Aurelia Visa, Montse Bazaga-García, Pascual Olivera-Pastor, Duane Choquesillo-Lazarte, David Marrero-López, Aurelio Cabeza, Rosario M. P. Colodrero, Konstantinos D. Demadis

**Affiliations:** † Departamento de Química Inorgánica, Universidad de Málaga, Campus Teatinos s/n, Málaga 29071, Spain; ‡ Crystal Engineering, Growth and Design Laboratory, Department of Chemistry, University of Crete, Voutes Campus, Crete GR-71003, Greece; § Romanian Academy, “Coriolan Dragulescu”, Institute of Chemistry, Timisoara 300223, Romania; ∥ Laboratorio de Estudios Cristalográficos, IACT, CSIC-Universidad de Granada, Granada 18100, Spain; ⊥ Departamento de Física Aplicada I, Universidad de Málaga, Campus Teatinos s/n, Málaga 29071, Spain

**Keywords:** coordination polymer, phosphonate, imidazole, proton conductivity, metal phosphide, electrocatalyst

## Abstract

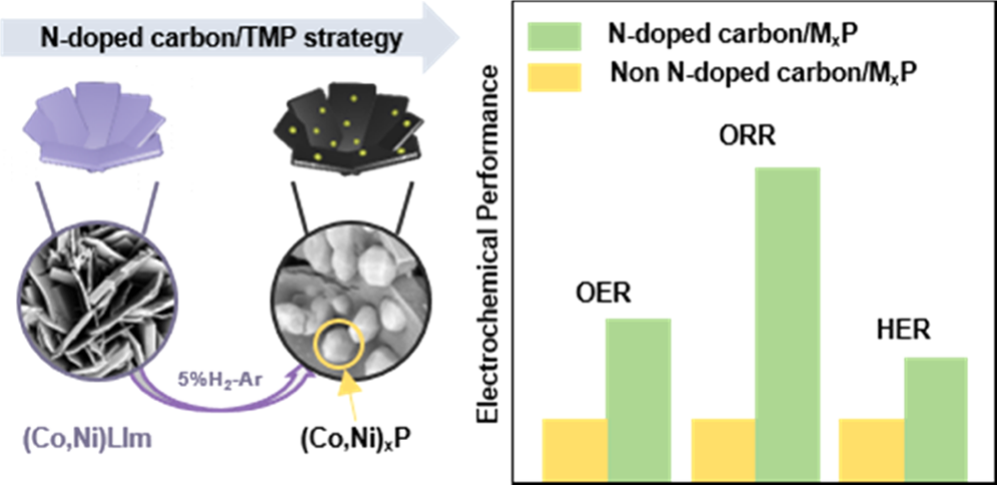

This work deals with the synthesis and characterization of one-dimensional (1D) imidazole-containing etidronates, [M_2_(ETID)(Im)_3_]·*n*H_2_O (M = Co^2+^ and Ni^2+^; *n* = 0, 1, 3) and [Zn_2_(ETID)_2_(H_2_O)_2_](Im)_2_, as well as the corresponding Co^2+^/Ni^2+^ solid solutions, to evaluate their properties as multipurpose materials for energy conversion processes. Depending on the water content, metal ions in the isostructural Co^2+^ and Ni^2+^ derivatives are octahedrally coordinated (*n* = 3) or consist of octahedral together with dimeric trigonal bipyramidal (*n* = 1) or square pyramidal (*n* = 0) environments. The imidazole molecule acts as a ligand (Co^2+^, Ni^2+^ derivatives) or charge-compensating protonated species (Zn^2+^ derivative). For the latter, the proton conductivity is determined to be ∼6 × 10^–4^ S·cm^–1^ at 80 °C and 95% relative humidity (RH). By pyrolyzing in 5%H_2_–Ar at 700–850 °C, core–shell electrocatalysts consisting of Co^2+^-, Ni^2+^-phosphides or Co^2+^/Ni^2+^-phosphide solid solution particles embedded in a N-doped carbon graphitic matrix are obtained, which exhibit improved catalytic performances compared to the non-N-doped carbon materials. Co^2+^ phosphides consist of CoP and Co_2_P in variable proportions according to the used precursor and pyrolytic conditions. However, the Ni^2+^ phosphide is composed of Ni_2_P exclusively at high temperatures. Exploration of the electrochemical activity of these metal phosphides toward the oxygen evolution reaction (OER), oxygen reduction reaction (ORR), and hydrogen evolution reaction (HER) reveals that the anhydrous Co_2_(ETID)(Im)_3_ pyrolyzed at 800 °C (CoP/Co_2_P = 80/20 wt %) is the most active trifunctional electrocatalyst, with good integrated capabilities as an anode for overall water splitting (cell voltage of 1.61 V) and potential application in Zn–air batteries. This solid also displays a moderate activity for the HER with an overpotential of 156 mV and a Tafel slope of 79.7 mV·dec^–1^ in 0.5 M H_2_SO_4_. Ni^2+^- and Co^2+^/Ni^2+^-phosphide solid solutions show lower electrochemical performances, which are correlated with the formation of less active crystalline phases.

## Introduction

Metal phosphonates represent a rich area of coordination and materials chemistry that has seen impressive growth in the last few decades^1−3^ due to their multiple properties as proton conductors,^4,5^ scale^6^ and corrosion inhibitors,^7^ catalysts,^8,9^ and as platforms for pharmaceutical drug delivery,^10,11^ among others. By combining (poly)phosphonic acid linkers^12,13^ and a variety of synthetic methodologies, including mechanochemical^14,15^ and high-throughput^16,17^ procedures, numerous metal phosphonates have been prepared for targeted applications, as they are amenable to establish appropriate structure/property relationships, assisted by advanced characterization techniques, such as synchrotron radiation^18^ or electron diffraction tomography.^19^

Recently, novel metal phosphonate compounds containing a secondary auxiliary ligand (SAL) have been synthesized.^20^ The incorporation of the SAL can serve several purposes: (a) alterations in framework dimensionality, (b) creation of hydrophobic domains, (c) changes in the coordination mode of the metal centers, (d) framework “decoration”, and, in certain cases, (d) rupture of the “coordination polymer” architecture. Apart from the common N-heterocyclic ligands, such as 2,2′-bpy, 1,10-phen, and 4,4′-bpy (bpy = bipyridine, phen = phenanthroline), other N-heterocycles have drawn attention because these may participate in proton transfer pathways, to increase the proton concentration in the material. The basic character of the N atom can act as a strong proton acceptor, thus forming protonic charge carriers.^21,22^

On the other hand, phosphorus-based materials, specifically transition metal phosphides (TMPs), have received considerable attention as promising electrocatalysts owing to their potential application in energy conversion and storage systems.^23−25^ Due to the uniform dispersion of the metal sites in metal phosphonates, only a one-step pyrolytic treatment is needed for the construction of TMPs, thus making them very attractive precursors of nonprecious metal catalysts (NPMCs) with interesting electrocatalytic properties. Moreover, heteroatom-doped carbon/TMP composites may also be generated by pyrolysis of phosphonate compounds with appropriate organic linkers. These heteroatoms, such as N, P, and S, could finely tune the electronic structure correspondingly.^26^ Taking this into consideration, the incorporation of a specific SAL on metal phosphonates can induce a modulation on the electronic density of the resulting pyrolyzed derivative and, therefore, improve the electrocatalytic performance.^26^

**Scheme 1:**
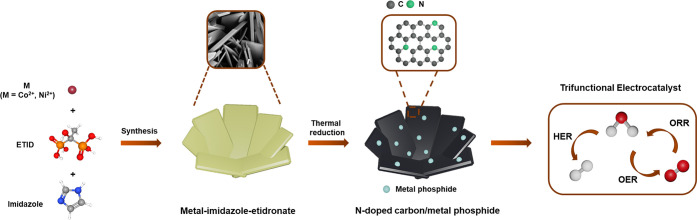
Schematic Illustration of the Preparation Process of Trifunctional N-Doped Carbon/TMP Electrocatalysts Derived from the Metal–Imidazole–Etidronates

Based on the aforementioned arguments, herein, we investigate the formation of metal phosphonates as precursors of TMPs with the ligand hydroxyethylidene-1,1-diphosphonic acid (etidronic acid, ETID) and imidazole as SAL, as part of a direct N-doping carbon strategy. A representative illustration of the synthetic strategy is shown in Scheme 1.

To date, only a few TMPs derived from metal phosphonates have been reported in the literature, and most of those have been selectively studied for the hydrogen evolution reaction (HER) due to their high electroactivity toward this reaction.^27^ Τhe N-doping strategy influences the carbon shell on these materials.^28^ However, N-doped TMPs generally exhibit poor electrochemical activity toward the oxygen evolution reaction (OER) and oxygen reduction reaction (ORR) due to the lack of active sites,^29^ presumably in the carbon shell, which precludes the access to multifunctional TMP electrocatalysts. Recently, electrocatalysts derived from ETID have been investigated toward the OER, ORR, and HER. For example, Maiti et al. obtained cobalt phosphosulfide nanoparticles dispersed into N,S,P-doped graphene with high ORR activity.^30^ Furthermore, the pyrolytic reduction of a Co-ETID at 750 °C yielded a mixture of cobalt phosphides (Co_2_P and CoP) with remarkable overpotentials in acidic, neutral, and basic media for the HER.^31^ On the other hand, bimetallic phosphonates may offer additional advantages in the electrochemical reactions due to the synergistic effect of the metals used.^32^ For instance, Fe/Ni phosphides dispersed into N,P-doped carbon nanosheets were obtained by one-step pyrolysis under an inert atmosphere and exhibited exceptional results toward the OER, ORR, and HER.^33^

In this paper, we report the syntheses, structures, and electrochemical behavior of several three-component metal–ETID–Im (metal = Co, Ni, Zn; ETID = etidronate; Im = imidazole) compounds. In these solids, the imidazole plays the role of either a terminal ligand or a counterion (protonated Im) to an anionic metal phosphonate framework. Five compounds with novel structural features are presented: {[Co_2_(ETID)(Im)_3_]·3H_2_O}*_n_* (**CoLIm-3**), {[Co_2_(ETID)(Im)_3_]·H_2_O}*_n_* (**CoLIm-1**), {[Co_2_(ETID)(Im)_3_]}*_n_* (**CoLIm-0**), {[Ni_2_(ETID)(Im)_3_]·3H_2_O}*_n_* (**NiLIm-3**), and {[Zn_2_(ETID)_2_(H_2_O)_2_](Im)_2_}*_n_* (**ZnLIm-2**) (L = ETID). Their syntheses, crystal structures, and proton conductivities are studied and discussed in detail. Moreover, the electrocatalytic behavior of diverse TMPs, obtained from Co^2+^, Ni^2+^, and their bimetallic phosphonate derivatives, are evaluated toward the OER, ORR, and HER.

## Experimental Section

### Reagents and Chemicals

All chemicals were purchased from Sigma-Aldrich and were used as received without further purification. Ion exchange column-deionized (DI) water was used for all syntheses. Stock solutions of NaOH and HCl were used for pH adjustment unless otherwise stated.

### Syntheses

#### Synthesis of [Co_2_(ETID)(Im)_3_]·3H_2_O (**CoLIm-3**)

A quantity of ETID (24 μL of a 60% aqueous solution, 0.1 mmol) was mixed with 10 mL of DI water. To that solution, imidazole (0.054 g, 0.8 mmol) and Co(NO_3_)_2_·6H_2_O (0.029 g, 0.1 mmol) were added under vigorous stirring. The pH was adjusted to 7.7. The final solution was left undisturbed for 7 days at room temperature. Purple crystals were formed and isolated by filtration, washed with a small amount of DI water, and air-dried. The yield was 75% based on the metal salt.

#### Synthesis of [Co_2_(ETID)(Im)_3_]·H_2_O (**CoLIm-1**)

A quantity of ETID (120 μL of a 60% aqueous solution, 0.5 mmol) was mixed with 10 mL of DI water, and then imidazole (0.272 g, 4.0 mmol) and Co(NO_3_)_2_·6H_2_O (0.1455 g, 0.5 mmol) were added under vigorous stirring. The pH was adjusted to 7.7 with a 4 M NH_3_ stock solution. The final solution was transferred to a 30 mL glass vial and then heated by microwave-assisted synthesis (Anton Paar Monowave 300 microwave) at 80 °C for 30 min under constant magnetic stirring (600 rpm). Alternatively, compound **CoLIm-1** was also prepared by refluxing the reactants following the same procedure described above. In both cases, purple polycrystalline solids were formed and isolated by filtration, washed with DI water, and air-dried. The yield was 70% based on the metal salt.

#### Synthesis of [Co_2_(ETID)(Im)_3_] (**CoLIm-0**)

Compound **CoLIm-0** was also prepared by heating under reflux, following a procedure similar to that of **CoLIm-1** but without magnetic stirring. The yield was 70% based on the metal salt.

#### Synthesis of [Ni_2_(ETID)(Im)_3_]·3H_2_O (**NiLIm-3**)

This compound was synthesized using a methodology similar to that of **CoLIm-1**, using Ni(NO_3_)_2_·6H_2_O as the metal source, under three different conditions: (a) hydrothermally, in an acid digestion bomb system equipped with a Teflon liner (80 °C for 3 days), (b) by microwave-assisted synthesis, at 80 °C for 30 min and constant magnetic stirring (600 rpm), or (c) under reflux, using the same conditions as in (b). For all cases, the final pH of the mixture was adjusted to 6.6. The first synthesis yielded green single crystals with a small amount of insoluble (unreacted) NiO, whereas the second and third syntheses afforded green single crystals together with the polycrystalline material of the desired product. The yield was 70% based on the metal salt.

#### Synthesis of [(Co*_x_*Ni_2–*x*_)(ETID)(Im)_3_]·*n*H_2_O (**(Co***_**x**_***Ni**_**2–*x***_**)LIm-*n***; *n* = 2, 3)

Solid solutions were obtained following the procedure described above for **CoLIm-1**, but varying the Co^2+^/Ni^2+^ molar ratios of the corresponding nitrate salts from 0.6 to 4.5 and maintaining the final solution pH fixed to 6.5.

#### Synthesis of [(Co_2–*z*_Ni*_z_*)(ETID)]·*n*H_2_O (**Co_2–*z*_Ni*_z_*L-*n***; *z* = 0, 0.6)

These metal etidronate precursors (without imidazole) were synthesized as “controls”, and were prepared as follows: M(NO_3_)_2_·6H_2_O (M = Co^2+^, Ni^2+^) and ETID (molar ratio = 2:1) were dissolved in DI water (10 mL) one by one under continuous stirring for 30 min, with a pH of around 1.2, and heated at 80 °C for water evaporation. The resultant products were subsequently dried at 60 °C in air overnight.

#### Synthesis of [Zn_2_(ETID)_2_(H_2_O)_2_](Im)_2_ (**ZnLIm-2**)

ETID (30 μL of a 60% aqueous solution, 0.125 mmol) and imidazole (0.017 g, 0.25 mmol) were mixed with 1 mL of DI water. The pH was adjusted to 2.5. Solid ZnO (0.005 g, 0.063 mmol) was added to the clear, colorless solution (but did not dissolve) and was finally placed into a sealed custom-made autoclave bomb, equipped with a Teflon liner. The bomb was placed in an oven and then heated at 80 °C for 3 days. After the solution was slowly cooled to room temperature, colorless crystals were isolated by filtration, washed with a small amount of DI water, and air-dried. The yield was 65% based on the metal salt.

#### Preparation of Metal Phosphides

The Co^2+^/Ni^2+^-phosphonate precursors were pyrolyzed under a 5%H_2_–Ar atmosphere at different temperatures. In a typical procedure, 60 mg of each precursor sample was placed in a ceramic boat inside a tubular furnace and purged with 5%H_2_–Ar. Afterward, metal phosphonates were calcined to selected temperatures for 2 h with a heating and cooling rate of 5 °C·min^–1^ under continuous gas flow (20 mL·min^–1^).

### Elemental, Thermal, and Microstructural Characterization

Attenuated total reflection-Fourier transform infrared (ATR-FTIR) spectra were collected on a Bruker VERTEX 70 optical spectrometer. Elemental analyses (C, H, N) were measured on a PerkinElmer 2400 analyzer. Thermogravimetric (TG) analysis (TGA) data were recorded on an SDT-Q600 analyzer from TA instruments. The temperature varied from room temperature (RT) to 900 °C at a heating rate of 10 °C·min^–1^ under air or N_2_ flow. Raman spectra were collected with a JASCO NRS-5100 Raman microscope using an excitation line of 532 nm (Nd:YAG laser), a power of 2.3 mW, and an acquisition time of 10 s. Scanning electron microscopy (SEM) was carried out on a FEI, Helios Nanolab 650, with an energy-dispersive X-ray spectrometer. High-angle annular dark-field scanning transmission electron microscopy (HAADF-STEM) and high-resolution transmission electron microscopy (HRTEM) were performed in a FEI Talos F200X. X-ray photoelectron spectroscopy (XPS) analyses were performed on a Physical Electronics ESCA 5701 spectrometer.

### Structural Characterization

Crystals for measurements were handled under inert conditions. They were manipulated while immersed in a perfluoropolyether protecting oil. Suitable single crystals were mounted on MiTeGen MicroMounts and subsequently used for data collection. X-ray diffraction data were collected with a Bruker D8 Venture diffractometer (Mo Kα radiation). The data were processed with the APEX3 suite.^34^ The structures were solved by an intrinsic method using SHELXT,^35^ which revealed the position of all nonhydrogen atoms. These atoms were refined on *F*^2^ by a full-matrix least-squares procedure using anisotropic displacement parameters.^36^ All hydrogen atoms were located in difference Fourier maps and included as fixed contributions riding on attached atoms with isotropic thermal displacement parameters 1.2 or 1.5 times those of the respective atom. In **CoLIm-3** and **NiLIm-3**, one imidazole ligand (N5) is disordered over two alternate positions in a ratio of 0:52:0.48 and 0.51:0.49, respectively.

Powder samples were analyzed by laboratory powder X-ray diffraction (PXRD) on a PANalytical EMPYREAN diffractometer in Bragg–Brentano or transmission configuration using Cu Kα_1,2_ radiation and a PIXcel detector. For **CoLIm-1** and **CoLIm-0** compounds, PXRD data were collected on a D8 ADVANCE (Bruker AXS) diffractometer equipped with a Johansson Ge(111) primary monochromator (Mo Kα radiation) and an energy-dispersive linear detector LYNXEYE XE. Their crystal structures were solved using the EXPO2014 program^37^ and the crystal structure of the **CoLIm-3** as a starting model. The crystal structures were optimized by the Rietveld method^38^ using the GSAS program^39^ and the graphic interface EXPGUI.^40^ The following soft constraints were imposed to preserve chemically reasonable geometries for the phosphonate, alkyl, and imidazole groups. The soft constraints were PO_3_C tetrahedron/P–O (1.53(1) Å), P–C (1.80(1) Å), O–O (2.55(2) Å), O–C (2.73(2) Å); alkyl group/C10OHC11H_3_ group/C10–C11 [1.50(1) Å], C10–OH [1.40(1) Å], P–OH [2.68(2) Å], C11–OH [2.40(2) Å]; and imidazole ring/C–C [1.40(1) Å], C–N [1.40(1) Å], C_ring_···C_ring_ [2.20(2) Å], C_ring_···N_ring_ [2.20(2) Å]. Furthermore, the structural disorder in the atomic position of the metal ions and imidazole rings of **MLIm-0** (M = Co, Ni) solids was modeled. No attempts to locate the H atoms were carried out due to the limited quality of the PXRD data. Final Rietveld plots are given in Figure S1. All crystallographic data are given in Table 1.

**Table 1:** Crystal Data for [M_2_(ETID)(Im)_3_]·*n*H_2_O (M = Ni^2+^ and Co^2+^; *n* = 0, 1, and 3) and [Zn_2_(ETID)_2_(H_2_O)_2_](Im)_2_

compound	**CoLIm-3**	**CoLIm-1**	**CoLIm-0**	**NiLIm-3**	**NiLIm-0**	**ZnLIm-2**
space group	*P*1̅	*P*1̅	*P*1̅	*P*1̅	*P*1̅	*P*2_1_
chemical formula	C_11_H_22_Co_2_N_6_O_10_P_2_	C_11_H_18_Co_2_N_6_O_8_P_2_	C_11_H_16_Co_2_N_6_O_7_P_2_	C_11_H_22_N_6_Ni_2_O_10_P_2_	C_11_H_16_N_6_Ni_2_O_7_P_2_	C_10_H_24_N_4_O_16_P_4_Zn_2_
formula mass (g·mol^–1^)	578.14	542.11	524.09	577.71	523.62	710.95
λ (Å)	0.71073	0.7093	0.7093	0.71073	1.5406	0.71073
*a* (Å)	10.1384(7)	11.5645(3)	10.7745(7)	10.0592(7)	10.6650(7)	7.7279(7)
*b* (Å)	10.3942(6)	9.7474(3)	9.7858(12)	10.3786(7)	9.8894(16)	14.7305(11)
*c* (Å)	10.7260(7)	8.9481(3)	9.9005(9)	10.6733(7)	9.8937(13)	10.8192(14)
α (deg)	79.087(2)	108.492(3)	118.426(4)	79.626(3)	117.938(5)	90
β (deg)	85.240(2)	92.555(3)	109.404(5)	85.776(3)	108.779(7)	99.588(5)
γ (deg)	66.411(2)	73.859(2)	90.807(7)	65.791(2)	90.842(9)	90
V (Å^3^)	1017.10(11)	917.83(6)	847.01(13)	999.69(12)	855.55(19)	1214.4(2)
crystal size (mm)	0.1 × 0.08 × 0.08			0.1 × 0.1 × 0.08		0.14 × 0.1 × 0.1
*Z*	2	2	2	2	2	2
ρ_calc_ (g·cm^–3^)	1.888	1.896	1.992	1.919	1.970	1.944
2θ range (deg)	2.170–27.587	3.00–50.00	2.00–55.00	2.402–27.525	3.00–90.00	2.357–27.556
data/restrains/parameters	4696/1/296	2404/58/130	2074/84/157	4550/1/303	4184/84/146	5568/1/333
no. reflections	30 881	3248	2222	18 013	821	27 575
independent reflections [*I* > 2σ(*I*)]	4696			4550		5568
GoF/Rf	1.079	2.78	6.44	1.059	2.65	1.024
*R* factor [*I* > 2σ(*I*)]	*R*_1_ = 0.0282,^a^ w**R**_2_ = 0.0631^a^			*R*_1_ = 0.0291,^a^ w*R*_2_ = 0.0662^a^		*R*_1_ = 0.0304,^a^ w**R**_2_ = 0.0737^a^
*R* factor (all data)	*R*_1_ = 0.0400,^a^ w*R*_2_ = 0.0671^a^			*R*_1_ = 0.0662,^a^ w**R**_2_ = 0.0705^a^		*R*_1_ = 0.0330,^a^ w*R*_2_ = 0.0753^a^
*R*_wp_		3.48	3.08		8.65	
*R*_p_		2.70	2.25		6.07	
CCDC code	2 043 945	2 088 939	2 117 781	2 043 946	2 099 218	2 043 950

a *R*_1_(*F*) = ∑||*F*_o_| – |*F*_c_||/∑|*F*_o_|; w*R*_2_(*F*^2^) = [∑w(*F*_o_^2^ – *F*_c_^2^)^2^/∑*F*^4^]^1/2^.

Thermodiffractometric data for **NiLIm-3**, **CoLIm-1**, and **ZnLIm-2** were obtained on samples loaded on an Anton Paar HTK1200N Camera, under an inert atmosphere, on a PANalytical X’Pert Pro automated diffractometer with Cu Kα_1_ and the X’Celerator detector. Data were collected from room temperature up to 900 °C with a heating rate of 5 °C·min^–1^ and a delay time of 5 min to ensure thermal stabilization.

### Electrochemical Measurements

Proton conductivity of the samples was determined by electrochemical impedance spectroscopy (EIS) on cylindrical pellets (diameter of ∼5 mm and thickness of ∼1.02–1.20 mm) obtained by pressing ∼38 mg of sample at 250 MPa for 1 min. The pellets were coated on both faces with a silver conductive paste (Sigma-Aldrich) and placed inside a temperature- and humidity-controlled chamber (Espec SH-222). EIS data were acquired with an HP4284A impedance analyzer or AUTOLAB PGSTAT302N equipped with a frequency response analyzer (FRA 32M) module over the frequency range from 20 Hz to 1 MHz with an AC applied voltage between 0.35 and 0.5 V. The pellets were first preheated (0.2 °C·min^–1^) from 25 to 80 °C and 95% relative humidity (RH) to ensure equilibrium with the atmosphere, and EIS data were recorded on cooling using a stabilization time of 3–5 h at each measurement temperature. Water condensation on samples was avoided by first reducing the relative humidity before decreasing the temperature. The measurements were automatically controlled with winDETA^41^ or NOVA 2.1.5^42^ software. For all compounds, the total pellet resistance (*R*_T_) was obtained from the analysis of the spectra using the ZView^43^ program.

The electrochemical activity of the catalysts was performed using a BioLogic VSP-128 potentiostat/galvanostat/impedance analyzer. The electrochemical tests were measured in a typical three-electrode configuration using a 5 mm diameter glassy carbon rotating disc electrode (GCE, 0.196 cm^2^) as a working electrode and a platinum rod and Ag/AgCl (3 M KCl) as counter and reference electrodes, respectively. The working electrode was prepared by mixing 4 mg of catalysts and 1.3 mg of carbon black (Super P conductive, <99%, Alfa Aesar) into 470 μL of ethanol/H_2_O (v/v = 1/1) with 30 μL of 5 wt % Nafion solutions, followed by sonication for 30 min to obtain a homogeneous ink. Then, 5 μL of the catalyst ink was drop-coated on the GCE and dried at room temperature to achieve a catalyst loading of 0.20 mg·cm^–2^. For comparison purposes, commercial RuO_2_ (Alfa Aesar, 99.9%) and 20 wt % Pt/C (HISPEC 3000, Johnson Matthey Company) were prepared and characterized under identical conditions. For the stability tests, a hydrophobic carbon paper (1 × 1 cm^2^, AvCarb MGL370) with a catalyst loading of 1 mg·cm^–2^ was employed as the working electrode.

All current densities were normalized to the geometrical surface area of the electrodes. The measured potentials vs Ag/AgCl were converted and referenced to the reversible hydrogen electrode (RHE) according to the Nernst equation: *E*(RHE) = *E*(Ag/AgCl) + 0.205 + 0.059 pH. Polarization curves were *iR* compensated (95%) with respect to the ohmic resistance of the solution. Before each measurement, the corresponding electrolyte was previously bubbled for 30 min with N_2_ gas for the OER and HER, and O_2_ in the case of the ORR, and maintained over the whole measurement to ensure the gas saturation in the solution. The optimal flow was found to be 20 mL·min^–1^ to obtain optimal OER/ORR/HER electrocatalytic performance (Table S1).

For the OER, cyclic voltammograms (CVs) and linear sweep voltammograms (LSVs) were measured at a scan rate of 20 and 10 mV·s^–1^, respectively, in the potential window of 1.0–1.7 V in a 1.0 M KOH electrolyte with the RDE rotate at 1600 rpm. The overpotential was determined from the equation η = *E*(RHE) – 1.23. HER measurements were conducted in a 0.5 M H_2_SO_4_ electrolyte with RDE rotation at 2000 rpm. CV experiments were performed at a scan rate of 20 mV·s^–1^ in the potential window of −0.6 to 0 V, while LSV curves were measured with a scan rate of 10 mV·s^–1^ in the potential range from −0.7 to 0 V.

The Tafel slope was calculated according to the equation
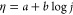
1where η, *b*, and *j* correspond to the overpotential, Tafel slope, and the current density, respectively. EIS measurements were carried out from 100 kHz to 0.1 Hz with an amplitude of 5 mV at a determined potential. The stability measurements were carried out by a continuous cycling test and a chronoamperometric (CA) response at their corresponding overpotential value.

ORR tests were conducted in a 0.1 M KOH electrolyte with the RDE rotating at 1600 rpm. CVs were measured at a scan rate of 20 mV·s^–1^ in the potential window from 0.2 to 1.2 V. LSVs were conducted at different rotation speeds (400–2025 rpm) under a scan rate of 10 mV·s^–1^ in the same potential range. The electron transfer number (*n*) for the ORR was determined according to the Koutecky–Levich (K–L) equation
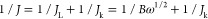
2
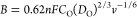
3where *J* represents the current density (A·cm^–2^), *J*_L_ and *J*_k_ are the diffusion-limiting and kinetic current density (A·cm^–2^), ω is the electrode rotation rate (rad·s^–1^), *F* is the Faraday constant (96 485 C·mol^–1^), *C*_O_ is the bulk concentration of O_2_ in 0.1 M KOH (1.2 × 10^–6^ mol·cm^–3^), *D*_O_ is the diffusion coefficient of O_2_ in 0.1 M KOH (1.9 × 10^–5^ cm^2^·s^–1^), and *v* is the kinematic viscosity of the electrolyte (1.09 × 10^–2^ cm^2^·s^–1^). Note that the constant 0.62 is employed with the rotation speed expressed in rad·s^–1^. The stability measurements were carried out by CA at 0.5 V, and the response was also evaluated under the addition of methanol.

### Water Splitting

Overall, water splitting was carried out on an AUTOLAB PGSTAT302N electrochemical workstation in 1.0 M KOH. A two-electrode homemade water-splitting electrochemical cell was assembled using **CoLIm-0@800** as symmetrical electrode materials and as an anodic electrode combined with a Pt/C cathodic electrode. Nafion 117 was used as a proton exchange membrane. The catalyst inks were drop-dried on carbon paper (1 × 1 cm^2^) with a loading mass of 1.0 mg·cm^–2^.

The theoretical amount of O_2_ gas was calculated from Faraday’s law
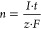
4where *n* is the number of moles, *I* is the current, *t* is the time, *z* is the transfer of electrons (for O_2_*z* = 4), and *F* is the Faraday constant.

The experimental amount of O_2_ gas was evaluated from the water displacement method by Dalton’s law
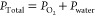
5assuming a vapor pressure of water of 21.1 mmHg at ambient conditions. The number of moles of oxygen gas produced in water displacement was calculated by
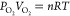
6where *V*_O_2__ is the volume of O_2_ produced, *T* is the temperature, and *R* is the gas constant. Finally, the Faradaic efficiency was obtained using the following relation
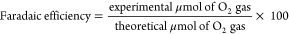
7

## Results and Discussion

### Syntheses

All syntheses were carried out in aqueous solutions, and the products were isolated as crystalline precipitates without the addition of any secondary precipitating solvent. According to the reported p*K*_a_ values of ETID,^44,45^ when conducting the syntheses at ∼pH 7.0, the phosphonic groups of the ETID ligand are completely deprotonated, and ETID carries a “4–” charge, requiring two coordinated divalent metal ions per ligand to obtain a neutral framework. Thus, the use of Co^2+^ and Ni^2+^ resulted in the formation of **MLIm-3** (M = Co, Ni) and **CoLIm-1**, with different water contents. In addition, an anhydrous phase of Co^2+^ (**CoLIm-0**) could be directly prepared by refluxing the reactants without stirring. In the case of **ZnLIm-2**, synthesized at pH = 2.5, the phosphonate ligand is tris-deprotonated and the imidazole is present as a positively charged imidazolinium cation. The purity of the bulk polycrystalline samples was confirmed by PXRD and elemental analysis (Table S2 and Figure S2).

Three monophasic bimetallic compositions were also isolated and later used to evaluate the electrochemical properties of their pyrolyzed derivatives. Their crystal structures, as determined by Le Bail fit (Table S3 and Figure S3), indicate that these compositions correspond to solid solutions, **(Co**_**x**_**Ni**_**2–*x***_**)LIm-*n*** (*n* = 2, 3). Depending on the Co^2+^/Ni^2+^ molar ratio, the resulting bimetallic solids crystallize with variable water contents and, thus, different crystal structures. Solids with *x* ≤ 1.2 are isostructural with **MLIm-3** (M = Co, Ni); while for *x* > 1.2, the compound displays the crystal structure of **CoLIm-1**.

### ATR-FTIR Studies

The ATR-FTIR spectra and the band assignments for the selected synthesized solids are given in Figure S4 and Table S4, respectively. The vibrational frequencies between 2600 and 3200 cm^–1^ are attributed to N–H stretching vibration and C–H symmetric and antisymmetric stretching vibrations of the heteroaromatic ring of imidazole.^46^ Τhe spectral region 900–1200 cm^–1^ is complex and includes several characteristic vibrations related to the −PO_3_ moieties of ETID.^47^ The other frequencies between 1440 and 1650 cm^–1^ are assigned to the C=C and C=N stretching vibrations of the heterocyclic aromatic ring.^46^ The range 700–850 cm^–1^ is associated with out-of-plane bending of C–H of heterocyclic rings of imidazole.^47^

### Crystal Structures

#### [M_2_(ETID)(Im)_3_]·3H_2_O (**MLIm-3**, M = Co, Ni)

**Figure 1:**
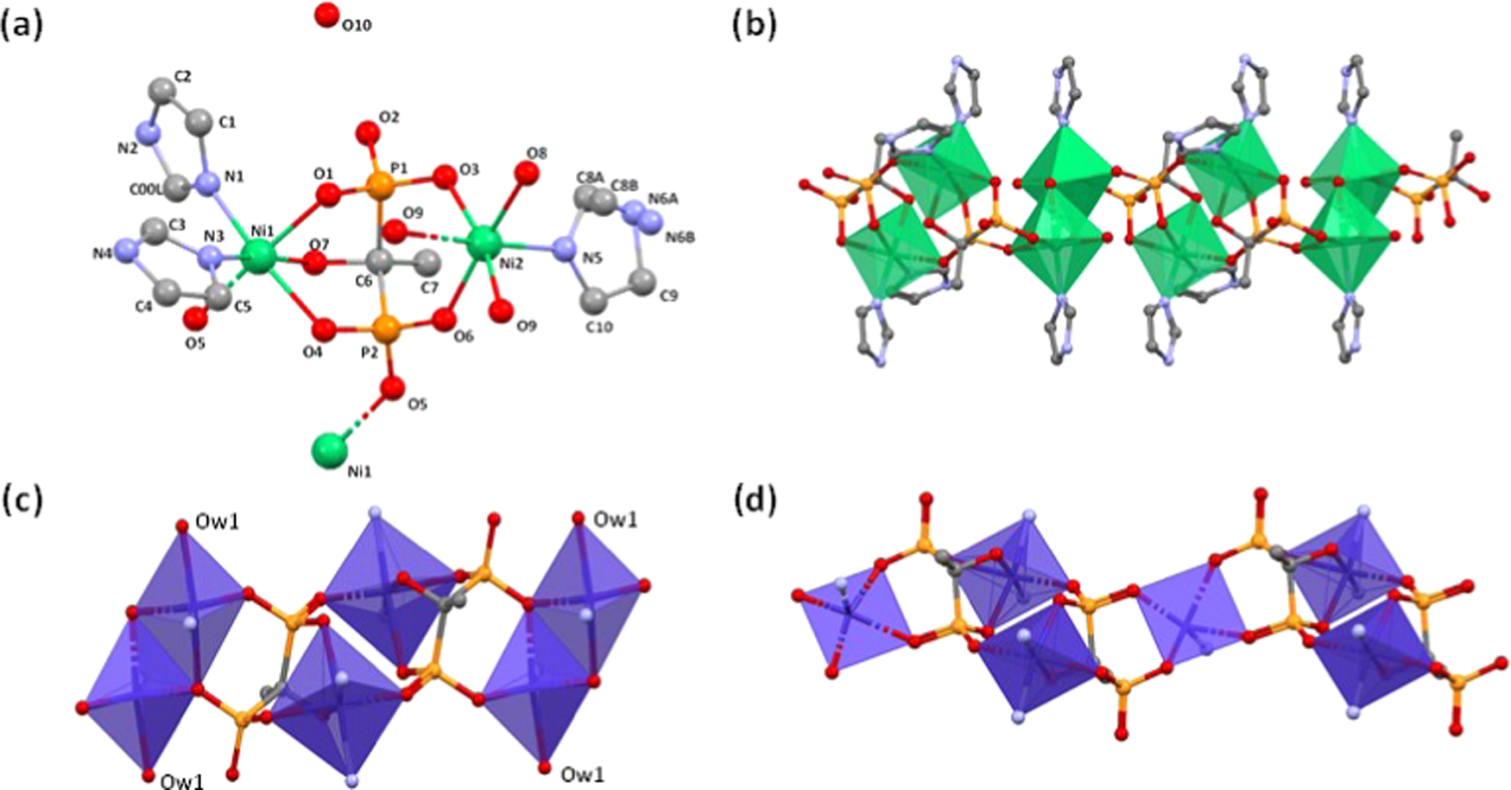
(a) Extended asymmetric part of the unit cell for 1D **MLIm-3** (M = Co, Ni) with atom labeling. (b) View of the chains along the *a*-axis for **MLIm-3** (M = Co, Ni). (c) Details of the two cobalt coordination environments for **CoLIm-1**, showing the dimeric trigonal bipyramidal and isolated octahedral modes. (d) Square pyramidal and octahedral coordination environments of the metal ions in 1D **MLIm-0** (M = Co, Ni). For clarity, imidazole rings, hydrogen atoms, and structural disorder are omitted, where appropriate.

The Co^2+^ and Ni^2+^ derivatives are isostructural. The structure of **CoLIm-3**, as a representative example, is shown in Figure 1. The compound crystallizes in the triclinic system with space group *P*1̅. The arrangement of atoms leads to a one-dimensional (1D) polymeric structure. Each asymmetric unit contains two kinds of Co^2+^ ions, one coordinated ETID molecule, three Co-coordinated imidazole molecules, two coordinated water molecules, and one lattice water molecule. Both metal ions are found in octahedral coordination geometry but surrounded by different ligand atoms.

Specifically, the Co1 ion is coordinated with two nitrogen atoms (N1, N3) from imidazole, three oxygen atoms (O1, O3, O7) from the phosphonic groups (P1, P2) of the ETID ligand, and one oxygen atom (O4) from the hydroxyl group of the ETID ligand. Metal coordination of the −OH of the ETID ligand is uncommon but not unprecedented.^48,49^ It is augmented by the chelating effect induced by the two adjacent phosphonate groups. Two neighboring Co1 centers are bridged by two phosphonate moieties (P1), which utilize the O1 and O3 oxygens, thus creating two Co–O–P–O–Co bridges. Each bridge is unsymmetrical as the Co1–O1 (2.064 Å) and Co1–O3 (2.202 Å) are not equal.

The Co2 ion is coordinated with one nitrogen atom (N5) from the imidazole, two oxygen atoms (O2, O5) from the phosphonic groups (P1, P2) of the ETID ligand, one oxygen atom (O9) from a water molecule, and two oxygen atoms (O8) from other water molecules, which bridge another Co2 atom. Two neighboring Co2 centers are bridged symmetrically by two water molecules (O8), but the formed Co_2_O_2_ rhomb is asymmetric, with Co–O bond lengths of 2.152 and 2.446 Å.

#### [Co_2_(ETID)(Im)_3_]·H_2_O (**CoLIm-1**)

The monohydrate phase, **CoLIm-1**, presents the distinctive structural features shown in Figure 1. The loss of two water molecules causes a rearrangement of the structure where the dimers Co_2_O_2_ are still maintained but now formed by penta-coordinated edge-sharing polyhedra of Co2, which are interconnected through the octahedra of Co1. In this case, the oxygens of Co_2_O_2_ moieties belong to the phosphonate group P1.

The coordination environment of Co1 remains the same as in the compound **CoLIm-3**. However, the Co2 dimeric units have undergone the following structural changes: (a) the Co centers are trigonal bipyramidal, (b) no bridging waters are present, (c) one terminal water is present, and (d) the trigonal bipyramidal units are now edge-sharing (two phosphonate Os, bridging the two Co2 centers).

#### [M_2_(ETID)(Im)_3_] (**MLIm-0**, M = Co, Ni)

The compound **NiLIm-0**, obtained by dehydration of the trihydrated phase at 220 °C (Figure S5), is isostructural to the “as-synthesized” **CoLIm-0**. The crystal structure of the anhydrous **NiLIm-0** was solved from PXRD data. Here, the chains are formed by alternating square pyramidal M^2+^ polyhedral and M^2+^ octahedra, linked to each other via phosphonate bridging oxygens (Figure 1).

#### [Zn_2_(ETID)_2_(H_2_O)_2_](Im)_2_ (**ZnLIm-2**)

**Figure 2:**
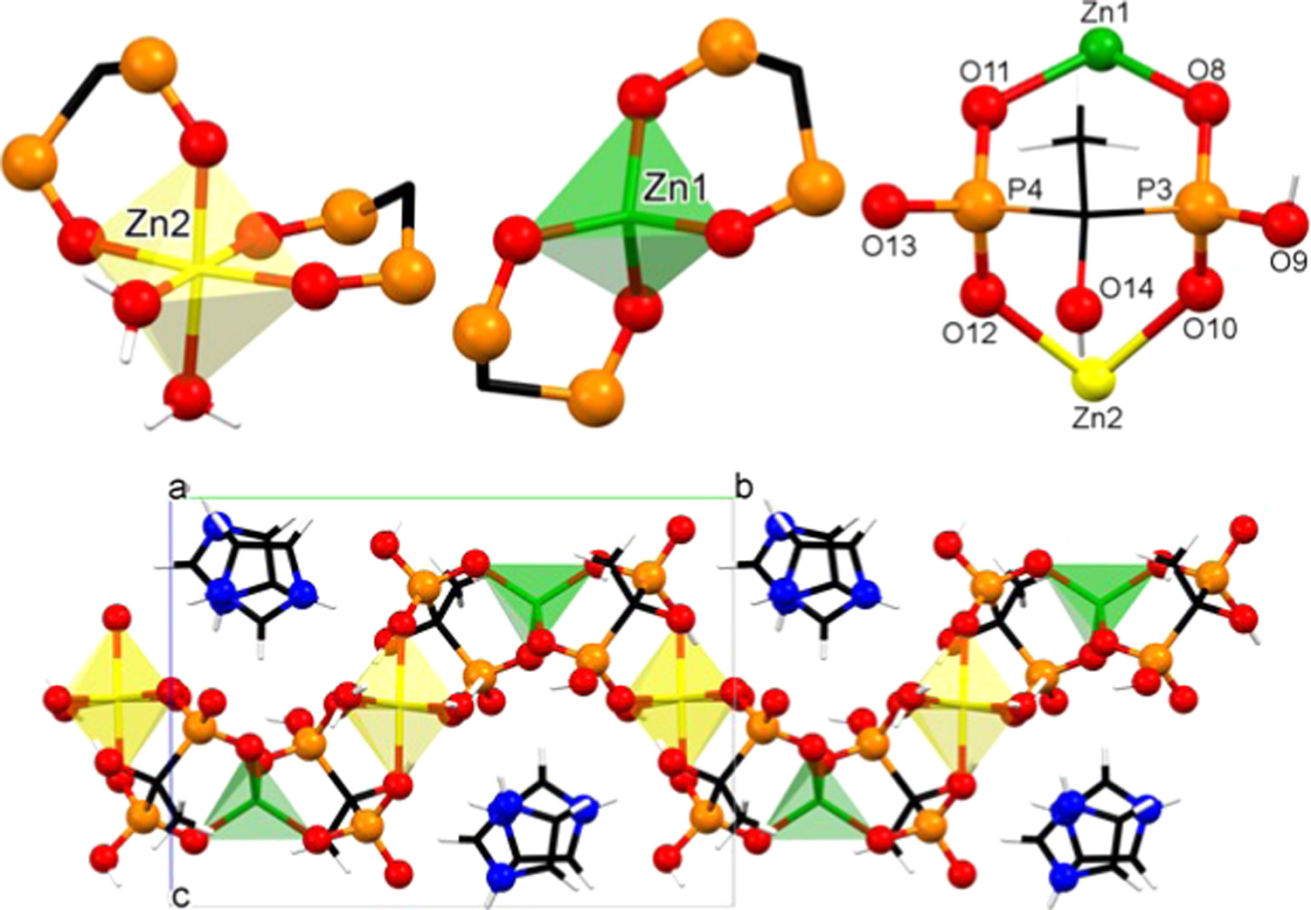
Structural features of **ZnLIm-2**.

The structure of **ZnLIm-2** is a 1D chain, as shown in Figure 2. The compound crystallizes in the monoclinic system (space group *P*2_1_). Each asymmetric unit contains two types of Zn^2+^ centers (one octahedral and one tetrahedral), two ETID ligands, two coordinated water molecules, and two protonated imidazole cations as counter ions. Zn1 is found in a tetrahedral coordination environment, and it is bound by four oxygens (O2, O6, O8, O12) from two different ETID ligands (P1, P2, P3, P4). The metal center Zn2 is found in an octahedral coordination environment. Specifically, Zn2 is coordinated by four phosphonate oxygens (O1, O5, O9, O14) from two different ETID ligands (P1, P2) and two oxygen atoms (O15, O16) from the bound water molecules.

The ETID ligand is tris-deprotonated. This means that one phosphonic acid group (P1) is singly deprotonated, whereas the other (P2) is doubly deprotonated. The “6–” total negative charge from the two ETID ligands is partially offset by the “4+” charge of the two Zn^2+^ cations. Their combination creates an anionic coordination polymer, with the negative charge of each unit compensated by the two imidazolium cations.

A similar, albeit structurally different, Zn^2+^ coordination polymer containing ETID and imidazolium cations, Zn_2_(ETID)(Im)_2_, has been recently reported in the literature.^50^ This three-dimensional (3D) compound only contains tetrahedrally coordinated Zn^2+^ and terminally bound imidazole.

### Thermal Behavior

The thermogravimetric analyses for Co^2+^, Ni^2+^, and Zn^2+^ derivatives are shown in Figure S6. The TG curves for **MLIm-3** (M = Co, Ni) display a slightly different thermal behavior, with the first weight-loss step of **CoLIm-3** starting at a lower temperature (100 °C). This weight loss corresponds to the loss of all water molecules (experimental: 9.7 wt %, found: 9.5 wt %). The second weight-loss step (∼28 wt %) starts at ∼220 °C, and it is due to the decomposition of the ETID and imidazole molecules. The monohydrate phase **CoLIm-1** presents a similar weight-loss profile for dehydration/decomposition, with the first weight loss of 3.9 wt % (calcd 3.3 wt %), in accordance with the loss of a water molecule. Compound **ZnLIm-2** shows a unique behavior, displaying three weight-loss processes. The first occurs in the 25–200 °C range, with 5.5 wt % weight loss (calcd 5.1 wt %). It is ascribed to the removal of two water molecules producing a crystalline anhydrous phase; however, its PXRD pattern could not be indexed (Figure S7). The subsequent weight-loss steps, ∼20.5 wt % between 250 and 600 °C, may be attributed to the decomposition of the organic components. Thermal decomposition (in N_2_) of **NiLIm-3** and **CoLIm-n** leads to Ni_2_P_2_O_7_ (PDF 01-074-1604) and a mixture of Co_2_P_2_O_7_ (PDF 01-070-1491) and Co_2_P (PDF 01-089-3030), respectively.

### Proton Conductivity

**Figure 3:**
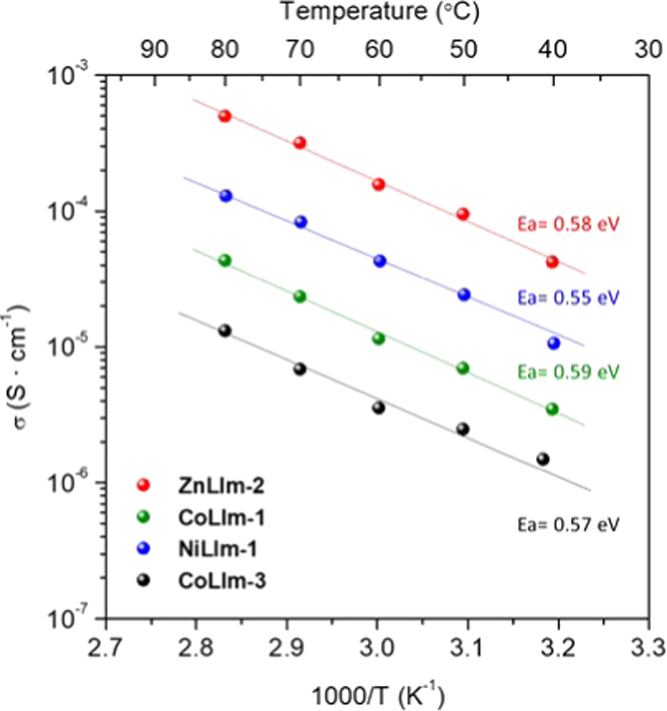
Arrhenius plots of the proton conductivity at 95% RH.

As reported for other compounds, the presence of imidazole molecules may assist in the creation of extended and well-defined proton-conducting pathways via H-bonding.^22,51^ However, the synthesized solids present low proton conductivities (Figures 3 and S8), except for **ZnLIm-2** (∼6 × 10^–4^ S·cm^–1^ at 80 °C and 95% RH). This can be related to the low pH used in the synthesis, facilitating the protonation of the phosphonate groups. The presence of phosphonic acid groups (−PO_3_H^–^ and/or −PO_3_H_2_) is a key factor for the creation of more extended H-bonding networks (Tables S5–S7) in metal phosphonate chemistry and, therefore, higher proton mobility. The conductivity values are comparable to other metal etidronates^52,53^ and the activation energy values (*E*_a_), which range from 0.55 to 0.59 eV, are indicative of a vehicle proton transport mechanism.^54^

### Electrocatalyst Characterization

The preparation of TMPs was conducted by one-step H_2_ thermal reduction of the corresponding metal phosphonate precursors in the temperature range of 550–850 °C (Figure S9). In the case of **NiLIm-3**, the reduction at 550 and 600 °C led to a mixture of crystalline and amorphous compounds, where two nickel phosphides were identified: Ni_3_P (PDF 01-074-1384) and Ni_12_P_5_ (PDF 01-074-1381). At higher temperatures, from 700 to 850 °C, only the hexagonal phase Ni_2_P (PDF 01-089-2742) was observed. Comparatively, higher pyrolytic temperatures were needed to obtain the cobalt phosphides; otherwise, phases with lower electrocatalytic activity, such as M_2_P_2_O_7_, would be present. For **CoLIm-3**, a reduction at 825 °C is required to achieve a complete conversion to cobalt phosphides, namely, CoP (PDF 98-004-3249) and orthorhombic Co_2_P (PDF 01-089-3030). In addition, pyrolysis of **CoLIm-1** and **CoLIm-0** led to a mixture of CoP and Co_2_P, albeit at 800 °C. Pyrolysis of the bimetallic solids resulted in phosphide mixtures, the hexagonal phase NiCoP (PDF 01-071-2336), and/or single monophosphide phases, depending on the Co^2+^/Ni^2+^ molar ratios and pyrolysis conditions. For non-N-doped carbon control materials, **CoL@800** and **Co**_**1.4**_**Ni**_**0.6**_**L@700**, the crystalline fractions were identified to be a mixture of phases Co_2_P (47 wt %) and CoP (53 wt %), and NiCoP (∼96 wt %), respectively.

**Figure 4:**
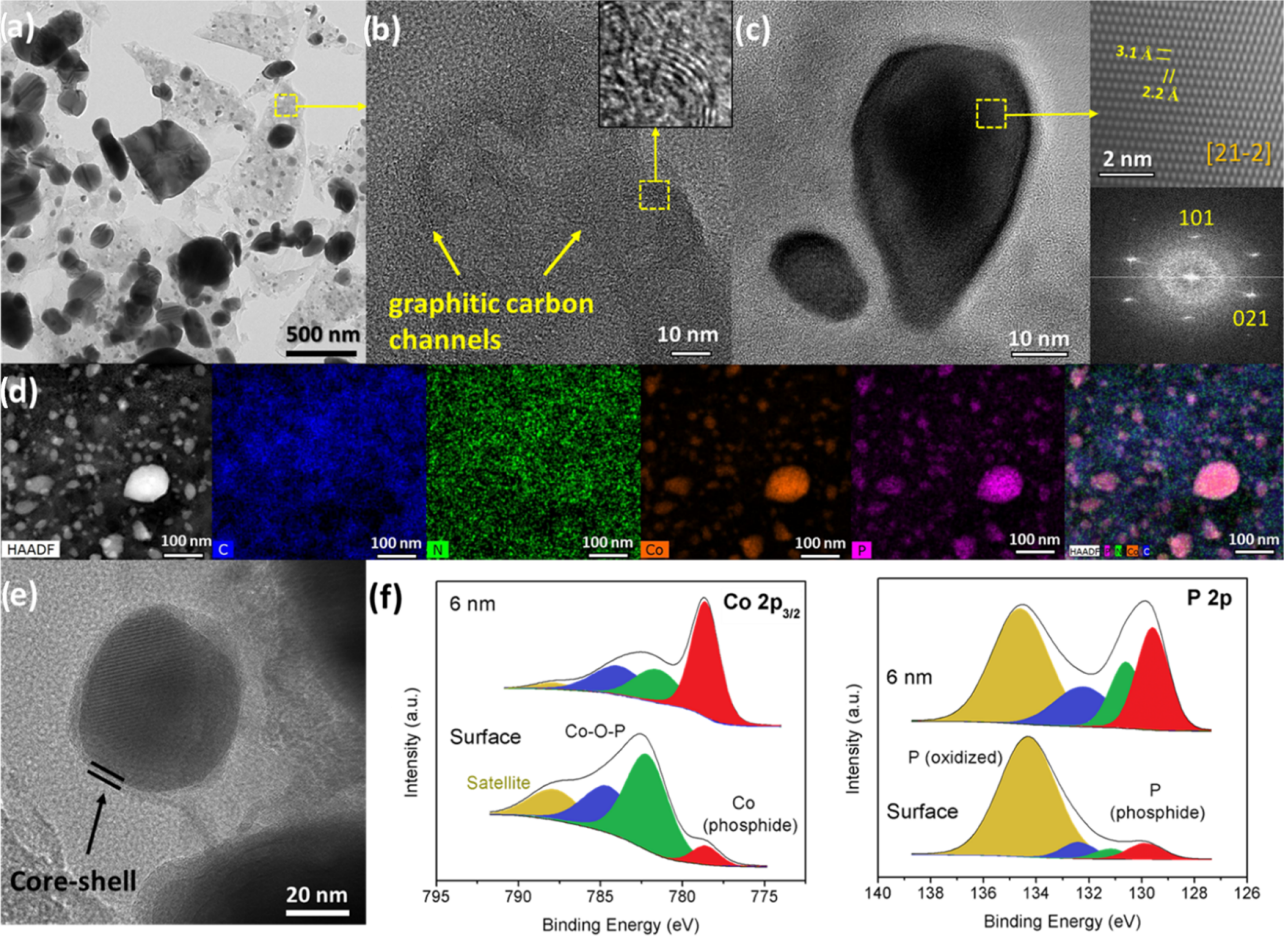
(a) TEM and (b, c) HRTEM images of sample **CoLIm-0@800** showing metal phosphide particles inside the graphitic carbon matrix and selected area electron diffraction (SAED) patterns of the Co_2_P phase. (d) HAADF-energy-dispersive X-ray (EDX) image and elemental distributions of C (blue), N (green), Co (orange), and P (purple) for **CoLIm-0@800**. (e) Detailed HRTEM image of the core–shell nanoparticles and (f) XPS spectra of **CoLIm-0@800** at different etching levels: Co 2p_3/2_ and P 2p regions.

Elemental analysis of the pyrolyzed samples (Table S8) indicates that the amount of carbonaceous material decreases considerably at temperatures higher than 800 °C. The Raman spectra (Figure S10) in the D and G band region showed two nitid signals at 1352 and 1583 cm^–1^ with practically identical D/G intensity ratios, suggesting that the extent of defects in the carbonaceous residue^55^ of these materials is quite similar. SEM images showed that thin rectangular micrometric particles, characteristic of the precursor metal etidronates, transform gradually into quasi-spherical nanometric particles featuring metal phosphide phases (Figures S11 and S12a). As expected, increasing the temperature of the reduction process gave rise to the size increase of the metal phosphide particles (Figure S13), which is detrimental to the electrochemical properties. Thus, we focused on materials pyrolyzed in the range of 700–800 °C. In addition, TEM images show heterogeneity in size, with the smaller nanosized metal phosphide particles being embedded into a graphitic carbon matrix, Figure 4a,b. The interplanar distances for **CoLIm-0@800**, determined by the HRTEM image in the [212̅] zone axis (Figure 4c), are assigned to (101) and (021) planes of the Co_2_P phase. Similar features were also observed for **NiLIm-3@700**, identified as hexagonal Ni_2_P (Figure S14). Element mapping images, Figures 4d and S15, confirm a uniform distribution of C and N in the graphitic substrate, while Co/Ni and P are located exclusively in the metal phosphide nanoparticles. In addition, the homogeneous distribution of the metal ions indicates the formation of solid solutions for the bimetallic compositions (Figures S16 and S17). This metal ion distribution differs from that reported elsewhere,^29^ in which doping metal ions are dispersed throughout both the metal phosphide nanoparticles and the graphitic substrate.

XPS spectra for **CoLIm-0@800**, Figure 4f, displays two contributions in the Co 2p_3/2_ region, attributed to Co^2+^ ions in phosphide (778.9 eV) and phosphate (782.2 and 784.6 eV) environments, respectively.^25,31^ Spectra at different etching levels revealed more clearly the phosphide core in agreement with the X-ray diffraction data (Figure S9). In the P 2p spectrum, the peaks at 129.7 and 130.7 eV are assigned to Co–P bonds, while those shown at 134.3 eV are attributed to P–O bonds.^25,56^ For the case of **NiLIm-3@700**, similar metal–P and P–O signals were observed in the Ni 2p_3/2_ and P 2p regions, respectively (Figure S18).^57^ As shown elsewhere, the N 1s spectra (Figure S18) can be fitted into three N contributions: pyridinic-N (398.7 eV), pyrrolic-N (400.5 eV), and graphitic-N (402.9 eV),^58^ suggesting the formation of a N-doped carbon matrix surrounding the metal phosphide core, Figure 4e.

### Oxygen Evolution Reaction

**Figure 5:**
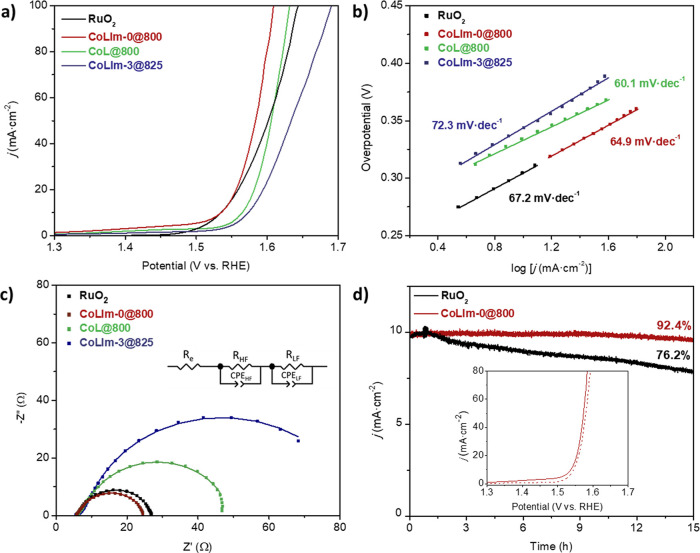
(a) LSV curves, (b) Tafel plots, and (c) EIS at 1.52 V of selected catalysts for the OER in 1.0 M KOH (inset: equivalent circuit used to fit the data). (d) Chronoamperometric response of **CoLIm-0@800** and RuO_2_ at their overpotential value, 300 mV (inset: LSV curves before (solid line) and after 500 cycles (dashed line) of CVs for **CoLIm-0@800**).

LSV curves were determined and compared to commercial RuO_2_ (Figures 5a and S19). As can be seen, the anhydrous phase **CoLIm-0** pyrolyzed at 800 °C, **CoLIm-0@800**, showed better performance (η_10_ = 298 mV) than the hydrated precursor phases (see Table 2). Lower η_10_ values correlate with an increasing content of the CoP phase, as determined by Rietveld analysis, in such a way that the maximum CoP/Co_2_P molar ratio (∼80/20 wt %) led to the most active electrocatalyst. The overpotential displayed by **CoLIm-0** is close to other reported metal phosphides (Table S9) and slightly lower than that determined for commercial RuO_2_ (308 mV). Comparatively, **CoL@800** displayed lower performance, which may be attributed to a lower concentration of the CoP phase (Table 2), which in turn may be influenced by the material history and the lack of the SAL, i.e., the absence of N in the carbon matrix.^29^

**Table 2:** Summary of the Crystalline Phases and Electrochemical Properties for Selected Catalysts

		OER		ORR		HER	
sample label	crystalline phases	η_10_ (mV)	Tafel slope (mV·dec^–1^)	*E*_onset_ (V)	*E*_1/2_ (V)	η_10_ (mV)	Tafel slope (mV·dec^–1^)
**NiLIm-3@700**	Ni_2_P^a^	321	55.2	0.78	0.66	185	88.2
**NiLIm-3@750**	Ni_2_P^a^	329	61.9	0.80	0.71	202	91.3
**CoLIm-3@825**	CoP (54%)	340	72.3	0.84	0.77	188	78.3
	Co_2_P^b^ (46%)						
**CoLIm-1@800**	CoP (74%)	298	68.6	0.85	0.77	162	78.6
	Co_2_P^b^ (26%)						
**CoLIm-0@800**	CoP (80%)	298	64.9	0.86	0.80	156	79.7
	Co_2_P^b^ (20%)						
**CoL@800**	CoP (47%)	329	60.1	0.80	0.67	183	80.6
	Co_2_P^b^ (53%)						

a Hexagonal phase.

b Orthorhombic phase.

In the case of nickel phosphides, **NiLIm-3@700** displayed the lowest overpotential (η_10_ = 321 mV), probably due to the smaller particles of Ni_2_P as well as the absence of other low activity nickel phosphides upon pyrolyzing at 700 °C. At higher pyrolysis temperatures, particle sintering gives rise to lower electrocatalytic activity (Figure S13 and Table S10).

The bimetallic derivatives, **Co**_**0.74**_**Ni**_**1.26**_**LIm@700**, **Co**_**1.2**_**Ni**_**0.8**_**LIm@700**, and **Co**_**1.64**_**Ni**_**0.36**_**LIm@800**, displayed higher η_10_ values (Table S10) compared to the monometallic phases. This trend was also observed for the control electrocatalysts. The absence of a Co^2+^/Ni^2+^ synergistic effect, as reported elsewhere,^59^ may be due to the formation of the less catalytically active phase, NiCoP (∼60–95%), together with CoP (∼5–40%).

The Tafel slope values (Figure 5b) for **CoLIm-0@800**, **CoL@800**, and **CoLIm-3@825** were 64.9, 60.1, and 72.3 mV·dec^–1^, respectively. The two first are around the theoretical value of 60 mV·dec^–1^, in which the rate-determining step is the chemical reaction after one-electron transfer reaction^59^ and are indicative of favorable reaction kinetics toward the OER upon comparing with the reference catalyst RuO_2_. The EIS data (Figure 5c) were fitted with the equivalent circuit displayed in the inset figure, which consists of a serial resistance *R*_e_ ascribed to the electrolyte solution with a value of approximately 5.5 Ω, regardless of the catalyst used. Two (R-CPE) elements, where R is a resistance in parallel with a constant phase element CPE, are needed to describe the electrode response adequately. The high-frequency process is usually related to surface porosity and exhibits a similar resistance value for the different catalysts (*R*_HF_ = 3 Ω).^60^ This contribution appears at a relaxation frequency of ∼100 Hz and has a capacitance value of 0.3 mF. The low-frequency semicircle, assigned to charge transfer, is the main contribution to the electrode polarization at a relaxation frequency of ∼10 Hz and has a capacitance similar to that of the high-frequency process. It is also worth noting that the charge-transfer resistance (*R*_ct_) of **CoLIm-0@800** (15.9 Ω) is comparable to that of commercial RuO_2_ (18.1 Ω) and significantly lower than that of **CoL@800** (32.2 Ω). In addition, **CoLIm-0@800** exhibits an improved durability compared to that of RuO_2_ for 15 h, with a current decay of only 7.6% for **CoLIm-0@800** against 23.8% for RuO_2_ (Figure 5d). Furthermore, **CoLIm-0@800** displayed high stability, as measured by LSV after 500 cycles of CV, Figure 5d (inset), as the overpotential only increased from 298 to 303 mV.

### Oxygen Reduction Reaction

**Figure 6:**
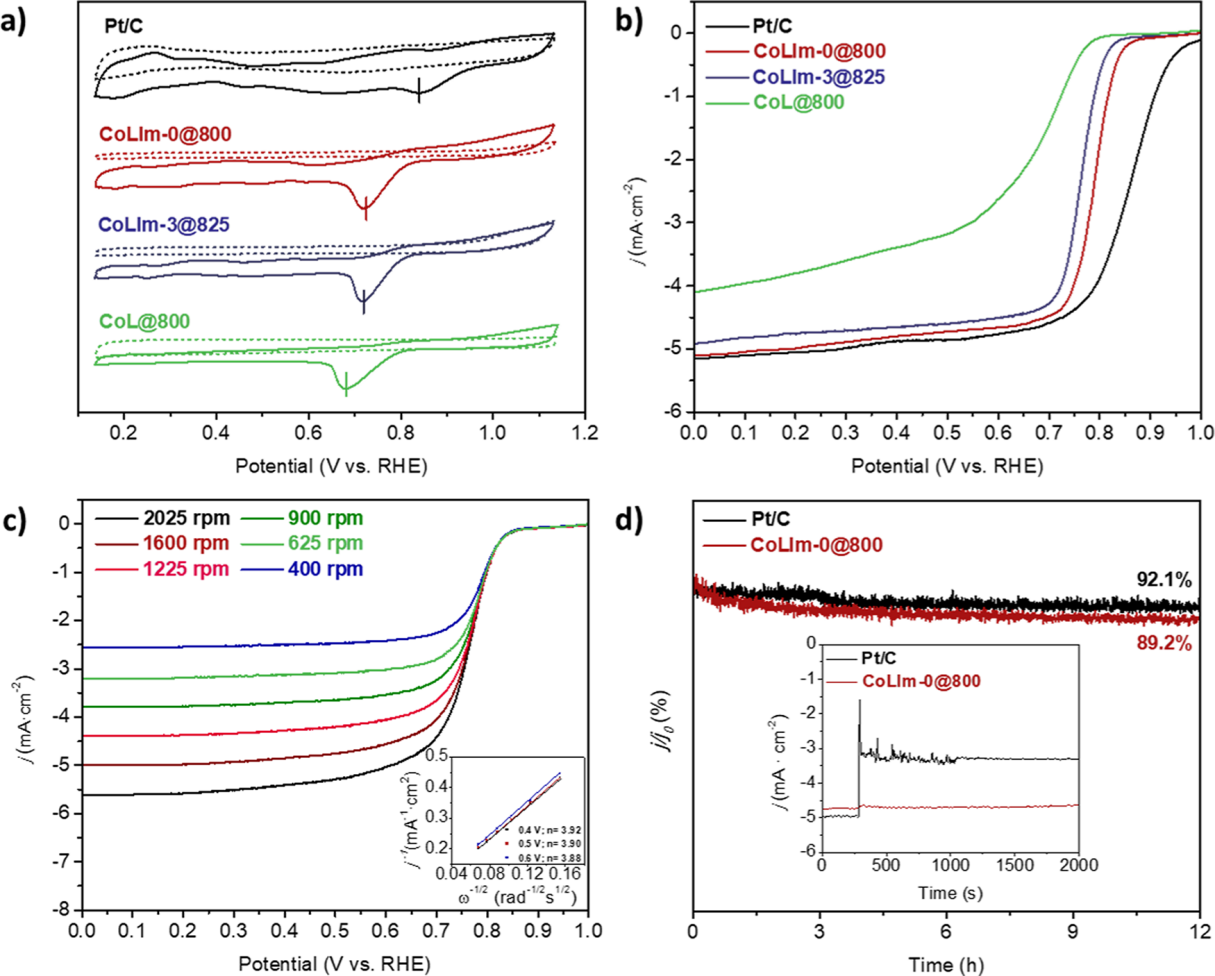
(a) CV curves in O_2_ (line) and N_2_ (dash)-saturated electrolyte and (b) LSV curves of selected catalysts in 0.1 M KOH. (c) LSV curves of **CoLIm-0@800** at different rotation speeds (inset: the K–L plot at different potentials). (d) Chronoamperometric response of **CoLIm-0@800** and Pt/C at 0.5 V (inset: CA measurement with the addition of methanol).

As observed for selected electrocatalysts, the CV curves (Figure 6a) show a significant oxygen reduction peak in O_2_-saturated 0.1 M KOH, which is not detected under N_2_. For **CoL@800**, the cathodic peak appears at 0.67 V, while this is shifted to 0.77 and 0.74 V for **CoLIm-0@800** and **CoLIm-3@825**, respectively, which are not far from that of the reference Pt/C catalyst (0.84 V) and is indicative of a high electrocatalytic ORR capability.

For this reaction, the catalyst performance trend was like that observed for the OER (Figures 6b and S20 and Table 2). Thus, **CoLIm-0@800** achieves the most positive onset potential (*E*_onset_) and half-wave potential (*E*_1/2_) with values of 0.86 and 0.80 V, respectively, close to those obtained for the Pt/C catalyst (0.98 and 0.86 V, respectively). Newly, the anhydrous precursor, **CoLIm-0**, led to better performance than that of the trihydrate (**CoLIm-3**) upon pyrolyzing at 800 °C. The catalysts derived from imidazole-containing precursors exhibited a significant enhancement of the ORR activity with respect to the control precursor, which can be related to a beneficial effect of a N-doped graphitic matrix formed when imidazole was used as SAL. Furthermore, N-doped carbon shell cobalt electrocatalysts displayed limiting current density (*j*_lim_) values, close to the value of the Pt/C benchmark electrocatalyst (∼−5 mA·cm^–2^).

Regarding the nickel derivatives, the highest activity was achieved for **NiLIm-3@750**, which showed slightly better properties than **NiLIm-3@700**, attributed to a higher reduction degree with an increase of the temperature. By increasing the cobalt content in the Ni_2_P framework, an enhancement of the ORR activity was observed up to a Co^2+^/Ni^2+^ molar ratio of 1.5 (**Co**_**1.2**_**Ni**_**0.8**_**LIm-3@750**). Higher cobalt molar ratios in the bimetallic compounds gave rise to the formation of a mixture of phases NiCoP (60 wt %) and CoP (40 wt %) in the solid **Co**_**1.64**_**Ni**_**0.36**_**LIm-2@800**, which resulted in a considerable improvement of the ORR activity, very close to that of the end member **CoLIm-1@800**, which may be ascribable to the presence of the highly active phase CoP (Table S10).

The LSV curves were measured under different rotation speeds, and the electron number transfer (*n*) per oxygen molecule was determined by applying the Koutecky–Levich (K–L) equation at different potentials (Figure 6c). The calculated *n* value was ∼3.9 for **CoLIm-0@800**, which is in agreement with a four-electron mechanism, indicative of high intrinsic activity and favorable reaction kinetics. CA measurements revealed high durability for **CoLIm-0@800**, after 12 h showing a behavior similar to that of the Pt/C catalyst. (Figure 6d). The CA test after the addition of methanol to the KOH electrolyte showed that **CoLIm-0@800** exhibits only a small oscillation in the current density, with a decay of 2.4%, recovering the electroactivity quickly and remaining stable during the CA test, Figure 6d (inset). Such behavior is in contrast with that of the Pt/C catalyst, which experiences a large oscillation in the current density, recovering only half of the current density. This result suggests that **CoLIm-0@800** exhibits favorable properties for applications such as direct methanol and alkaline fuel cells owing to its high ORR selectivity and strong methanol tolerance.^61^

### Hydrogen Evolution Reaction

**Figure 7:**
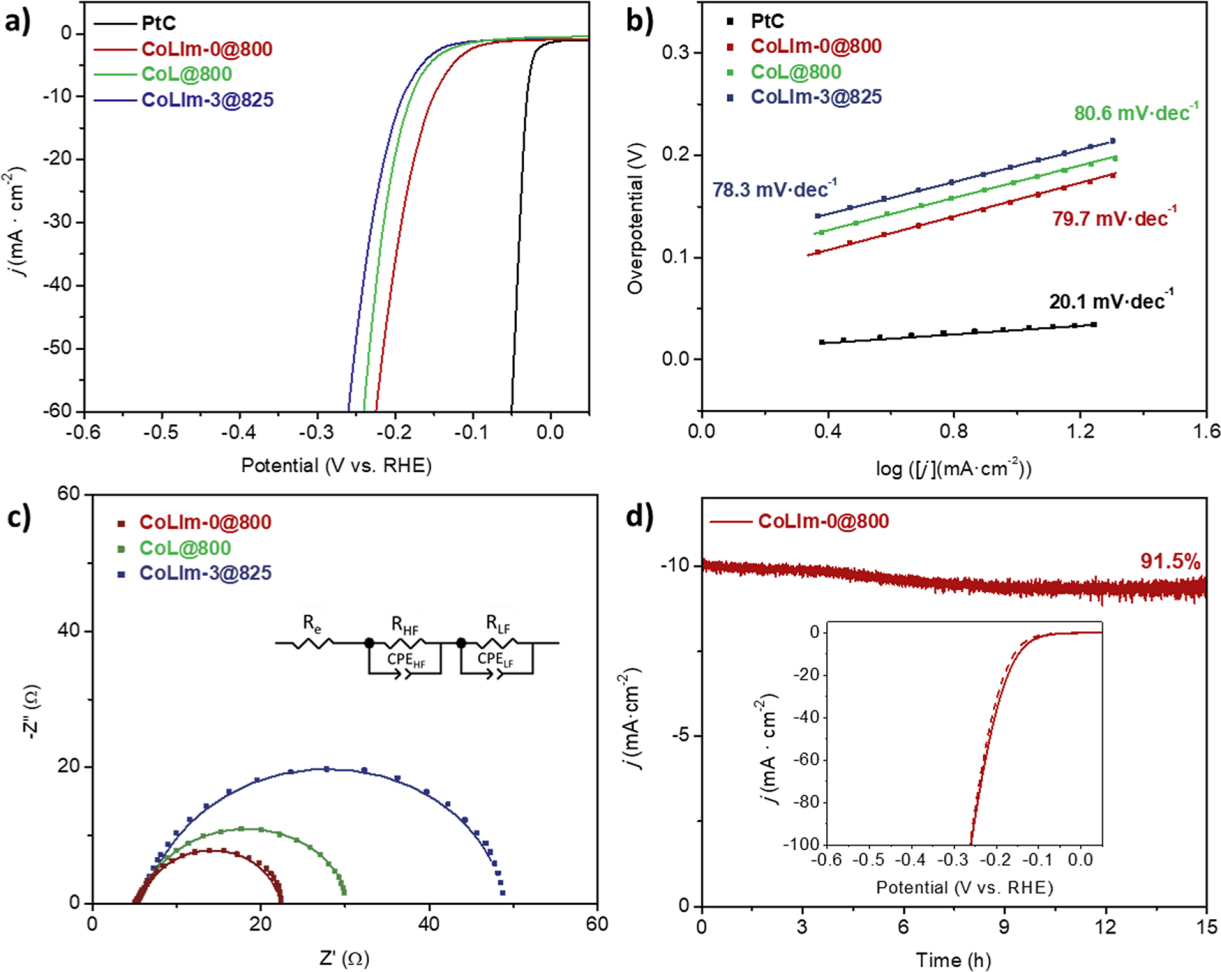
(a) LSV curves, (b) Tafel plots, and (c) EIS at −190 mV (inset: equivalent circuit) of selected metal phosphides for the HER in 0.5 M H_2_SO_4_. (d) Chronoamperometric response of **CoLIm-0@800** at −200 mV (inset: LSV curves before (solid line) and after 500 cycles (dashed line) of CVs for **CoLIm-0@800**).

The HER activity trend of the electrocatalysts is similar to that previously observed for the OER (Table 2). According to the LSV curves (Figures 7a and S21), the most active electrocatalyst was **CoLIm-0@800** (η_10_ = 156 mV) followed by **CoLIm-1@800** (η_10_ = 162 mV), **CoL@800** (η_10_ = 170 mV), **CoLIm-3@825** (η_10_ = 185 mV) and **NiLIm-3@700** (η_10_ = 188 mV), values that are far away from that observed for Pt/C (34 mV). Although the reactions were conducted in acidic conditions, the repetitive trend observed points to the conclusion that having a high concentration of the CoP phase and low sintering of the particles are the most beneficial features for high performance. The overpotential values for **CoLIm-0@800** and **NiLIm-3@700** fall within the range obtained with monophasic cobalt (CoP) and nickel (Ni_2_P) phosphides, respectively, embedded into N,P-codoped carbon backbones (Table S9).^25^

The Tafel slope values, Figure 7b, agree well with the Volmer–Heyrovský mechanism, suggesting an electrochemical hydrogen adsorption or discharge step (Volmer reaction) followed by an electrochemical desorption step (Heyrovský reaction).^62^ The overpotentials and Tafel slopes found are similar to those previously reported for metal phosphide electrodes (Table S9).

On the other hand, the low charge-transfer resistance for **CoLIm-0@800** (*R*_ct_ = 22.7 Ω), determined from the low-frequency semicircle of the Nyquist plots (Figure 7c), confirmed its better HER performance and faster proton discharge kinetics, compared to **CoL@800** and **CoLIm-3@825** (*R*_ct_ = 26.3 and 45.9 Ω, respectively). **CoLIm-0@800** also exhibited remarkable durability (Figure 7d) with a total current decay of 8.5% for 15 h of operation. In addition, the overpotential increased slightly from 156 to 164 mV after 500 CVs, Figure 7d (inset).

To evaluate the stability of **CoLIm-0@800**, Rietveld, SEM and XPS analyses were conducted after the durability tests. Calculated percentages of the crystalline CoP/Co_2_P phases from PXRD data (Figure S22) varied between 77:23 and 83:17, indicating small deviations from the initial electrocatalyst composition (Table 2). Also, SEM images (Figure S12) revealed no significant changes in morphology and particle size after durability tests. XPS showed only marginal changes in the surface composition of **CoLIm-0@800** after the HER test (Figure S23), while postreaction materials exposed to alkaline conditions (OER and ORR) experienced changes mainly in the Co 2p_3/2_ region due to the appearance of a new signal at 780.8 eV ascribable to CoO(OH).^63^ Interestingly, the signals in the P 2p region remain practically unchanged, pointing to the fact that the catalyst integrity is maintained after the electrochemical tests.

### Multifunctional Activity

To evaluate the electrochemical capability of the prepared catalysts, **CoLIm-0@800** was selected as the working electrode owing to its remarkable electrocatalytic properties toward the OER, ORR, and HER, comparable to other high-performance cobalt materials.^64−66^ Its bifunctional activity for electrochemical oxygen cycle (e.g., zinc–air battery) in alkaline electrolytes is highlighted, as the potential difference (Δ*E* = *E*_*j*=10_ – *E*_1/2_)^29^ between OER and ORR calculated for this electrode material, 0.74 V, is comparable to the values for the Pt/C (Δ*E* = 0.83 V) and the RuO_2_ (Δ*E* = 1.04 V) benchmark electrocatalysts.

**Figure 8:**
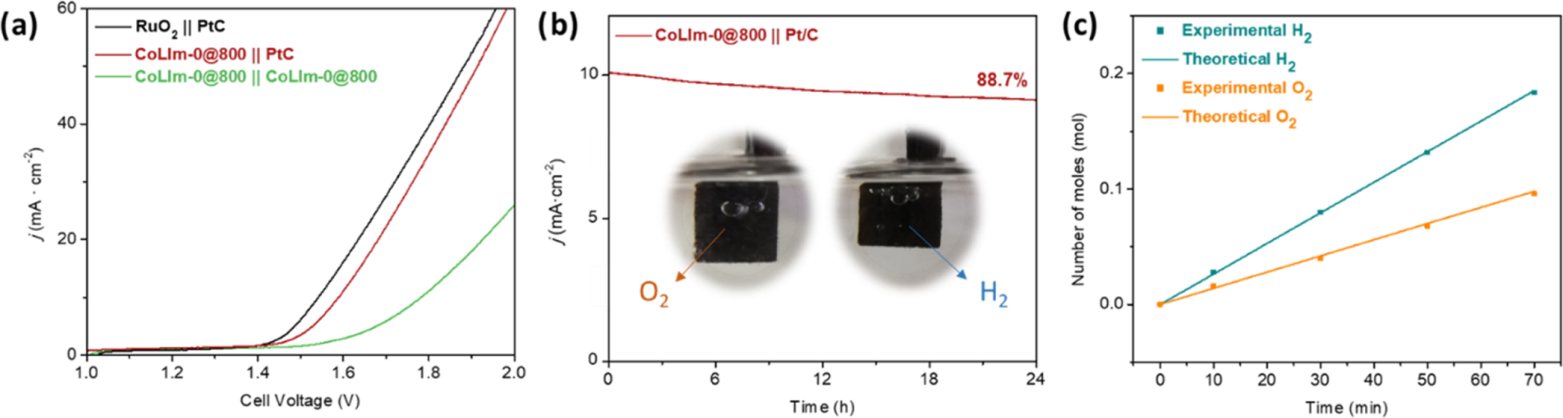
(a) Polarization curves of different electrodes tested for water splitting in 1.0 M KOH. (b) CA curve for **CoLIm-0@800C** working as an anode (the inset shows optical photographs inside the water-splitting cell). (c) Theoretical and experimental number of moles of gas generated.

To further study the electrochemical activity of **CoLIm-0@800**, experiments were carried out in an assembled alkaline water electrolyzer to test its capability for overall water splitting (Figures 8 and S24). Although **CoLIm-0@800** exhibits a modest behavior as a bifunctional catalyst, the cell voltage of 1.77 V at a current density of 10 mA·cm^–2^ is comparable to those reported for other nanosized cobalt phosphides.^67,68^ However, the system **CoLIm-0@800**||20 wt % Pt/C needs a cell voltage of 1.61 V, which is slightly higher than that of the RuO_2_||20 wt % Pt/C (1.56 V) and exhibits considerable durability, with a current density loss of 11.3% for over 24 h. The generated amounts of oxygen agree well with the theoretical value (Figure 8c), with a Faraday efficiency for the OER close to ∼98% after 70 min of operation. The required cell voltage falls within the range obtained for other reported materials based on cobalt-containing electrocatalysts.^69,70^ These results render **CoLIm-0@800** an adequate electrode material for water splitting, comparable with the expensive reference RuO_2_ electrocatalyst.

## Conclusions

A family of N-doped carbon/TMP (Co^2+^-, Ni^2+^- and Co^2+^/Ni^2+^) electrocatalysts has been prepared and tested as electrode materials for the OER, ORR, and HER. The electrochemical behavior was dependent on the metal phosphide composition, pyrolysis temperature, and particle size. The coordination environment of the metal ions in metal phosphonate precursors, which in turn is conditioned by the hydration degree, is a key factor in determining the TMP phase composition. We have also demonstrated that inserting a N-containing SAL, such as imidazole, is a convenient way to enhance the electrocatalytic properties. In addition, the presence of protonated imidazole in [Zn_2_(ETID)_2_(H_2_O)_2_](Im)_2_ gives rise to a moderate proton conductivity with a proton transfer vehicle mechanism. For cobalt electrocatalysts, a CoP/Co_2_P molar ratio of ∼4 (**CoLIm-0@800**) was found as the most active composition with characteristic η_10_ values of 298 mV (OER) and 156 mV (HER), and relatively high ORR performance (*E*_onset_ = 0.86 V and *E*_1/2_ = 0.80 V), as well as favorable kinetics and adequate stability. Furthermore, this trifunctional electrocatalyst exhibited a remarkable integrated capability as an anode for overall water splitting (cell voltage = 1.61 V). Although less active than the cobalt derivatives, the obtained nickel phosphide-based electrocatalysts displayed the best performance when the crystalline phase Ni_2_P was present. For these electrocatalyst systems, bimetallic Co^2+^/Ni^2+^ solid solutions could be prepared. Increasing the Co^2+^ content in the structure of Ni_2_P, up to a Co^2+^/Ni^2+^ molar ratio of 1.5, did not significantly improve the electrocatalytic behavior. However, a higher Co^2+^ enrichment in the bimetallic composition led to a mixture of CoNiP (60 wt %) and CoP (40 wt %) with a noticeable improvement of electrocatalytic activity attributed to the high proportion of the CoP phase.

## Supporting Information Available

Rietveld and Le Bail plots; PXRD patterns; ATR-FTIR; thermal analysis; thermodiffractometric studies; complex impedance plane plots; Raman analysis; SEM-EDX; TEM and HAADF-EDX images; XPS spectra; LSV curves; photographs of homemade water-splitting equipment; details of elemental analysis, crystallographic data, H-bond distances, and electrochemical properties for selected catalysts (PDF)
Crystallographic data files (ZIP)
