# Supplementary material for: Exploiting the Multifunctionality of M2+/Imidazole–Etidronates for Proton Conductivity (Zn2+) and Electrocatalysis (Co2+, Ni2+) toward the HER, OER, and ORR
Source: ACS Appl Mater Interfaces. 2022 Feb 22;14(9):11273–87. doi: 10.1021/acsami.1c21876 (PMC8915163; doi:10.1021/acsami.1c21876)
Supplement: Supplementary file 1 — am1c21876_si_001.pdf [file am1c21876_si_001.pdf]

Supporting Information

for

Exploiting multifunctionality of M^{2+}/imidazole-etidronates for proton conductivity (Zn^{2+}) and electrocatalysis (Co^{2+}, Ni^{2+}) toward HER, OER and ORR

Álvaro Vílchez-Cózar,[#] Eirini Armakola,[‡] Maria Gjika,[‡] Aurelia Visa,[§] Montse Bazaga-García,[#] Pascual Olivera-Pastor,[#] Duane Choquesillo-Lazarte,[⊥] David Marrero-López,[¥] Aurelio Cabeza,[#] Rosario M.P. Colodrero,[#] Konstantinos D. Demadis[*‡]*

[#] Departamento de Química Inorgánica, Universidad de Málaga, Campus Teatinos s/n, Málaga-29071, Spain.

[‡] Crystal Engineering, Growth and Design Laboratory, Department of Chemistry, University of Crete, Voutes Campus, Crete, GR-71003, Greece.

[§] Romanian Academy, "Coriolan Dragulescu" Institute of Chemistry, Timisoara, 300223, Romania.

[⊥] Laboratorio de Estudios Cristalográficos, IACT, CSIC-Universidad de Granada, Granada-18100, Spain.

[¥] Departamento de Física Aplicada I, Universidad de Málaga, Campus Teatinos s/n, Málaga-29071, Spain

Corresponding Author e-mail address:

*** Prof. Dr. K.D. Demadis, e-mail: demadis@uoc.gr.**

*** Dr. R.M.P. Colodrero, e-mail: colodrero@uma.es.**

Table of contents

Figures

Figure S1. Rietveld plots for (a) **CoLIm-1**, (b) **CoLIm-0** and (c) **NiLIm-0**.

Figure S2. Calculated (black) vs. experimental (red) PXRD patterns of compounds (a) **CoLIm-3**, (b) **NiLIm-3** and (c) **ZnLIm-2**.

Figure S3. Le Bail plots for (a) **$Co_{0.74}Ni_{1.26}$LIm-3**, (b) **$Co_{1.2}Ni_{0.8}$LIm-3** and (c) **$Co_{1.64}Ni_{0.36}$LIm-2**.

Figure S4. ATR-FTIR studies of selected metal-imidazole-etidronates.

Figure S5. Thermodiffractometric studies for (a) **NiLIm-3**, compared with as-prepared **CoLIm-0**, and (b) **CoLIm-1**.

Figure S6. TGA curves of **ZnLIm-2** (black), **CoLIm-1** (red), **CoLIm-3** (blue) and **NiLIm-3** (green).

Figure S7. Thermodiffractometric study for **ZnLIm-2.**

Figure S8. Nyquist plots for (a) **CoLIm-3**, (b) **CoLIm-1**, (c) **NiLIm-3** and (d) **ZnLIm-2** at 95% of relative humidity (RH) and different temperatures.

Figure S9. PXRD patterns of the resulting pyrolyzed derivatives from (a) **NiLIm-3**, (b) **CoLIm-3**, (c) **CoLIm-n** (n= 0, 1) and (d) **(Co_xNi_{2-x})LIm-n** (n= 2, 3) at different temperatures under 5%H_2-Ar atmosphere.

Figure S10. Raman spectra of selected metal phosphides.

Figure S11. SEM images for precursors (a) **CoLIm-0**, (b) **CoLIm-1**, (c) **CoLIm-3** and (d) **NiLIm-3**.

Figure S12. SEM images of (a) as-synthesized **CoLIm-0@800** and after conducting durability tests for (b) OER, (c) ORR and (d) HER.

Figure S13. SEM and TEM images for (a,b) **NiLIm-3@700** and (c,d) **NiLIm-3@800**.

Figure S14. (a) TEM image of **NiLIm-3@700** and HRTEM image of (b) the graphitic carbon matrix and (c) **NiLIm-3@700** particle (figures d and e shows the indexation of the electron diffraction pattern corresponding to Ni_2P nanoparticles).

Figure S15. HAADF-EDX image and elemental distributions of C, N, Ni and P for **NiLIm-3@700.**

Figure S16. (a) TEM and (b) HAADF-EDX images with elemental distribution of (c) C, (d) N, (e) Co, (f) Ni, (g) P and (h) C+N+Co+Ni+P for **$Co_{0.74}Ni_{1.26}$LIm-3@700**.

Figure S17. (a) TEM and (b) HAADF-EDX images with elemental distributions of (c) C, (d) N, (e) Co, (f) Ni, (g) P and (h) C+N+Co+Ni+P for **$Co_{1.64}Ni_{0.36}$LIm-2@800**.

Figure S18. XPS spectra of (a) Ni $2p_{3/2}$, (b) P 2p and (c) N 1s regions for **NiLIm-3@700.**

Figure S19. Oxygen evolution polarization curves of pyrolyzed materials derived from (a) **NiLIm-3**, (b) **CoLIm-n** (n= 0, 1, 3) and (c) **(Co_xNi_{2-x})LIm-n** (n=2, 3) compared with commercial RuO_2 in 1.0 M KOH.

Figure S20. Oxygen reduction polarization curves of pyrolyzed materials derived from (a) **NiLIm-3**, (b) **CoLIm-n** (n= 0, 1, 3) and (c) **(Co_xNi_{2-x})LIm-n** (n=2, 3) compared with Pt/C electrocatalyst in 0.1 M KOH.

Figure S21. Hydrogen evolution polarization curves of pyrolyzed materials derived from (a) **NiLIm-3**, (b) **CoLIm-n** (n= 0, 1, 3) and (c) **(Co_xNi_{2-x})LIm-n** (n=2, 3) compared with Pt/C electrocatalyst in 0.5 M H_2SO_4.

Figure S22. PXRD patterns after OER, ORR and HER tests of **CoLIm-0@800** compared to the as-prepared catalyst.

Figure S23. XPS spectra of Co $2p_{3/2}$ and P 2p regions after OER, ORR and HER tests of **CoLIm-0@800** compared to the as-prepared catalyst.

Figure S24. Photographs of (a) the homemade water splitting equipment and (b) gas production at different times (10 to 70 min. from left to right). Hydrogen is liberated at topside and oxygen at downside.

Tables

Table S1. Rietveld phase quantification and selected electrochemical properties for **CoLIm-0@800** after annealing in 5%H_2-Ar.

Table S2. Elemental analysis for **MLIm-n** (n=0, 1, 3) and **(Co_xNi_{2-x})LIm-n** (n=2, 3) compounds**.**

Table S3. Crystallographic data for **(Co_xNi_{2-x})LIm-n** (n=2, 3) compounds obtained by Le Bail fit**.**

Table S4. ATR-FTIR bands assignments for **CoLIm-1**.

Table S5. H-bond distances for **ZnLIm-2**.

Table S6. H-bond distances for **NiLIm-3.**

Table S7. H-bond distances for **CoLIm-3.**

Table S8. Elemental analysis for metal phosphides derived from **NiLIm-3**.

Table S9. Comparison of OER, HER and ORR performance of selected electrocatalysts with recent studies under similar electrochemical measurement conditions.

Table S10. Summary of the crystalline phases and electrochemical properties for selected electrocatalysts.

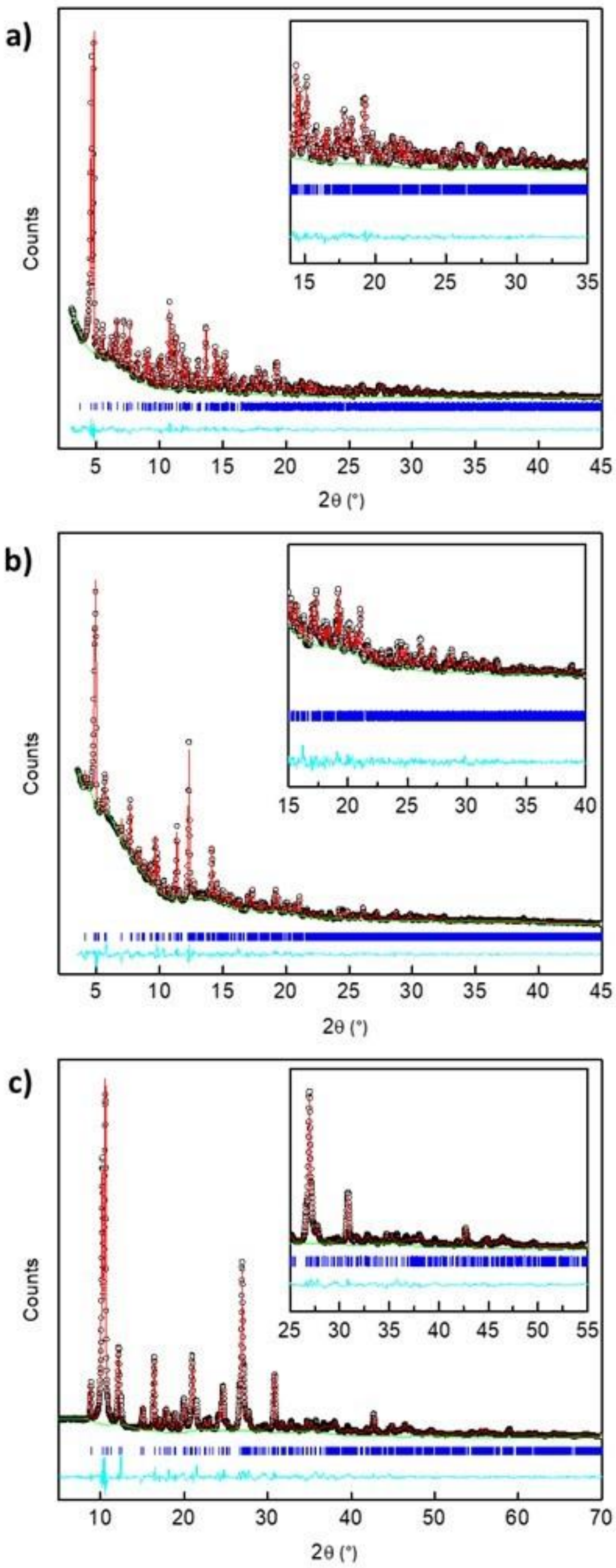

Figure S1. Rietveld plots for (a) **CoLIm-1**, (b) **CoLIm-0** and (c) **NiLIm-0**.

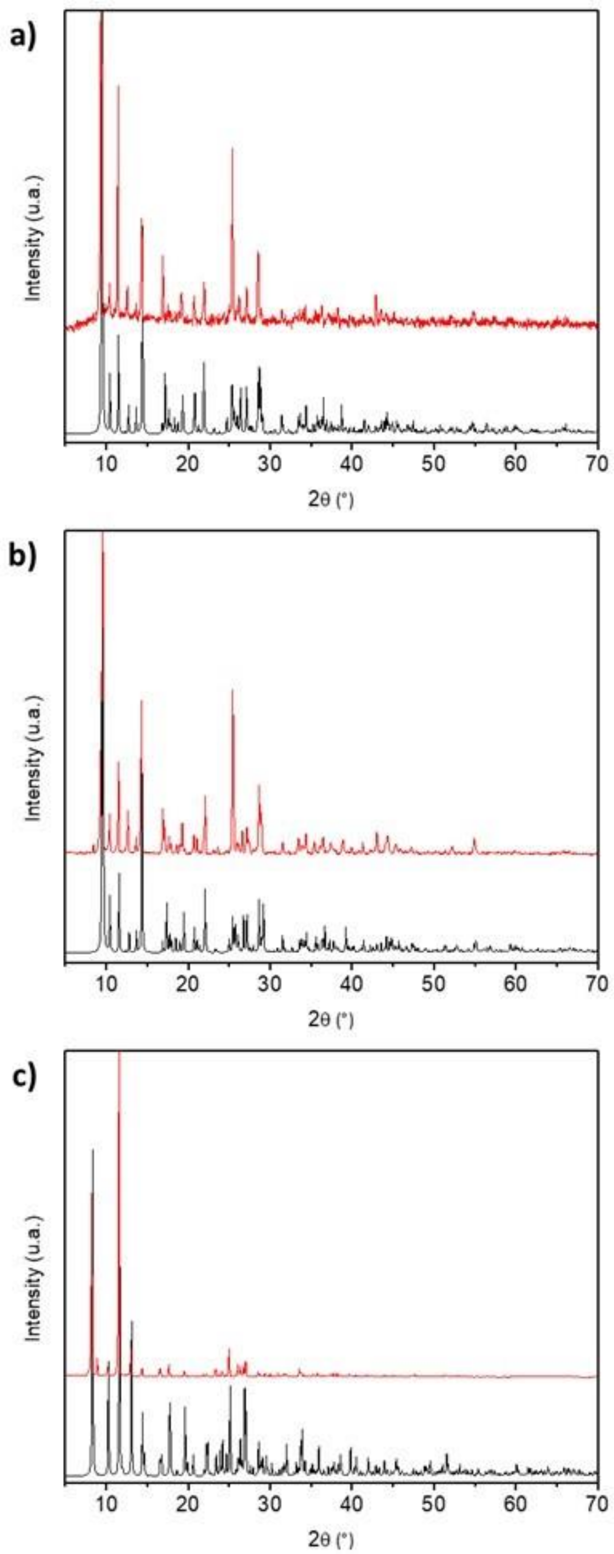

Figure S2. Calculated (black) vs. experimental (red) PXRD patterns of compounds (a) **CoLIm-3**, (b) **NiLIm-3** and (c) **ZnLIm-2**.

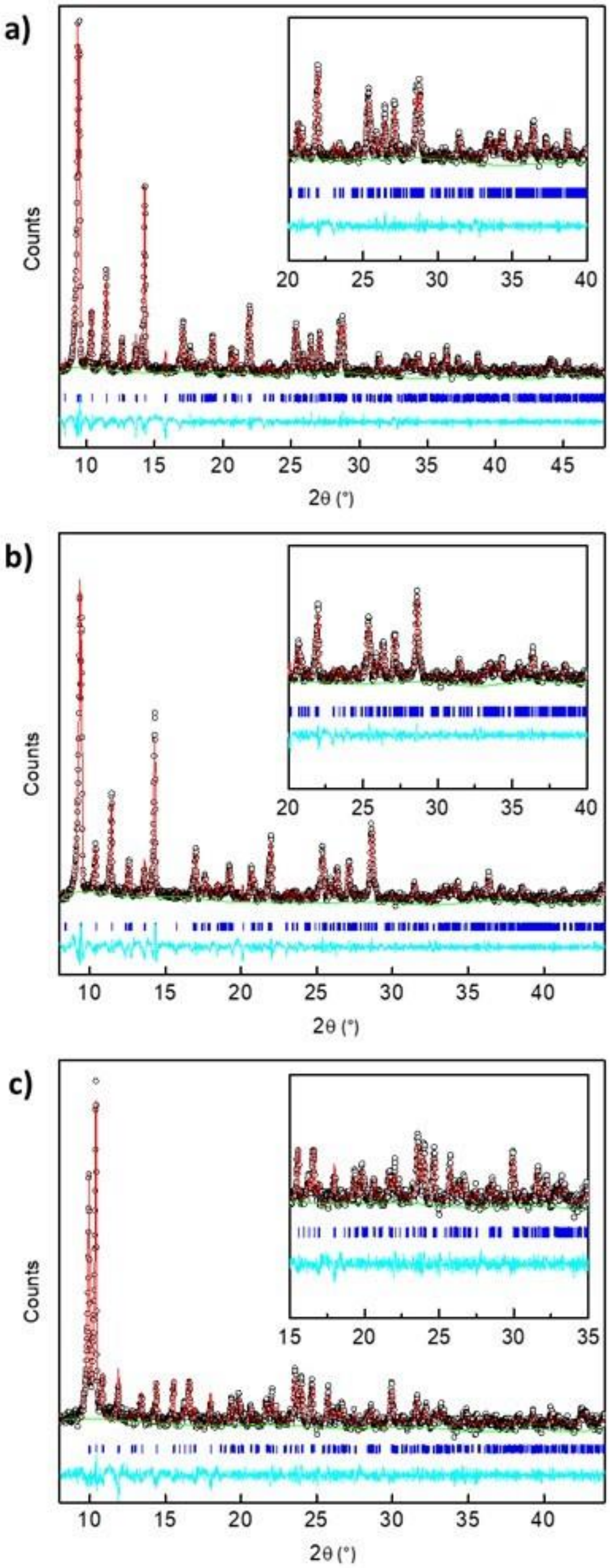

Figure S3. Le Bail plots for (a) **$Co_{0.74}Ni_{1.26}$LIm-3**, (b) **$Co_{1.2}Ni_{0.8}$LIm-3** and (c) **$Co_{1.64}Ni_{0.36}$LIm-2**.

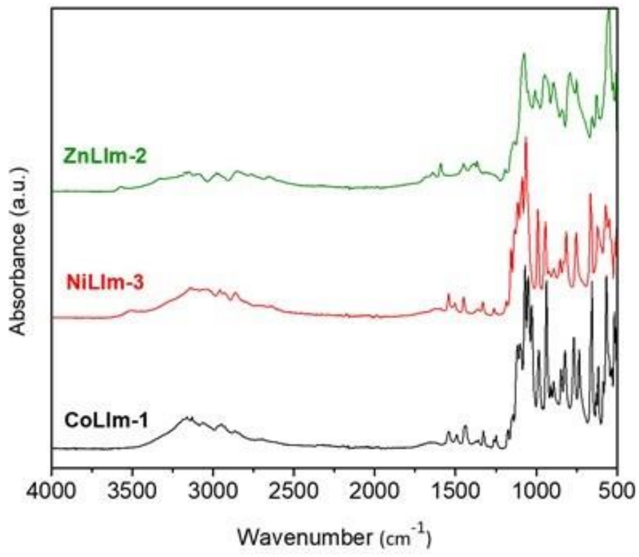

Figure S4. ATR-FTIR studies for selected metal-imidazole-etidronates.

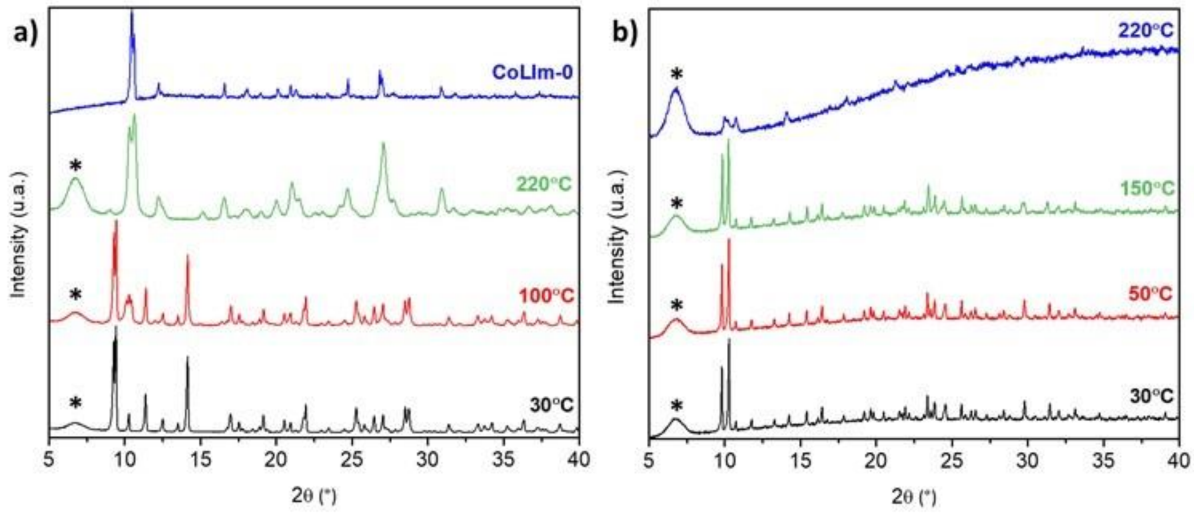

Figure S5. Thermodiffractometric studies for (a) **NiLIm-3**, compared with as-prepared **CoLIm-0**, and (b) **CoLIm-1** (stars highlight peaks coming from the chamber).

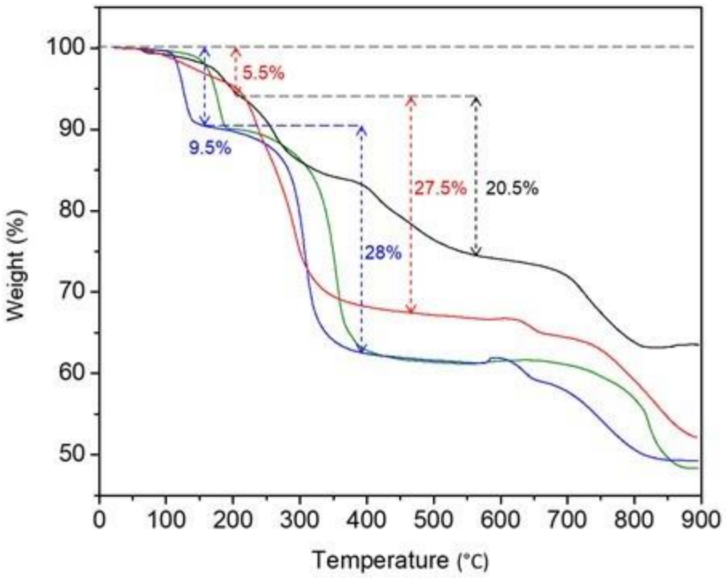

Figure S6. TGA curves of **ZnLIm-2** (black), **CoLIm-1** (red), **CoLIm-3** (blue) and **NiLIm-3** (green).

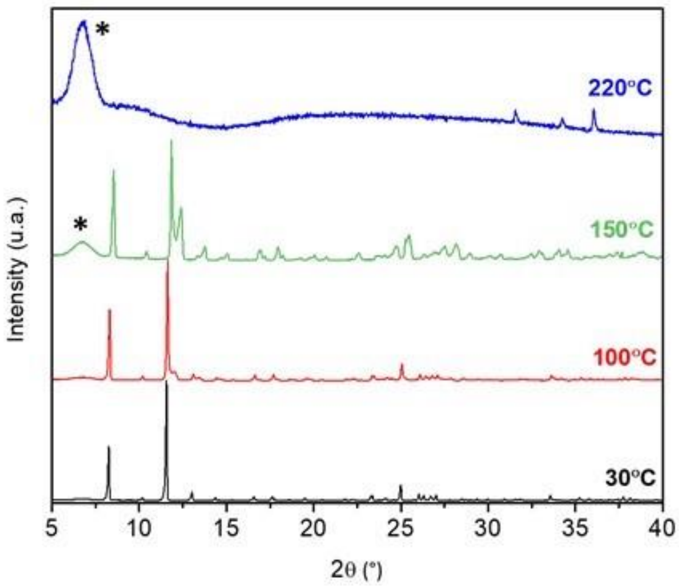

Figure S7. Thermodiffractometric study for **ZnLIm-2** (stars highlight peaks coming from the chamber).

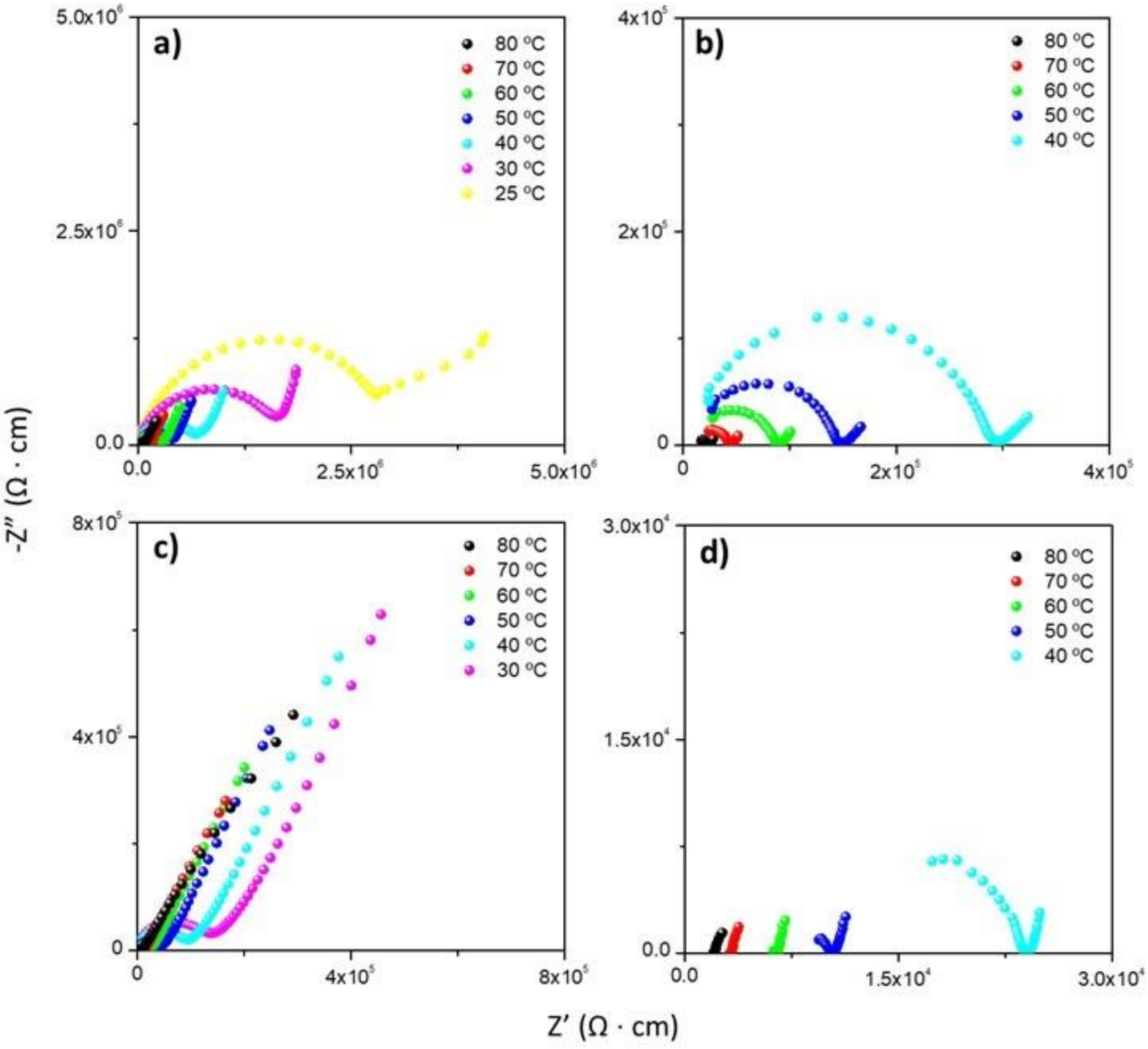

Figure S8. Nyquist plots for (a) **CoLIm-3**, (b) **CoLIm-1**, (c) **NiLIm-3** and (d) **ZnLIm-2** at 95% of relative humidity (RH) and different temperatures.

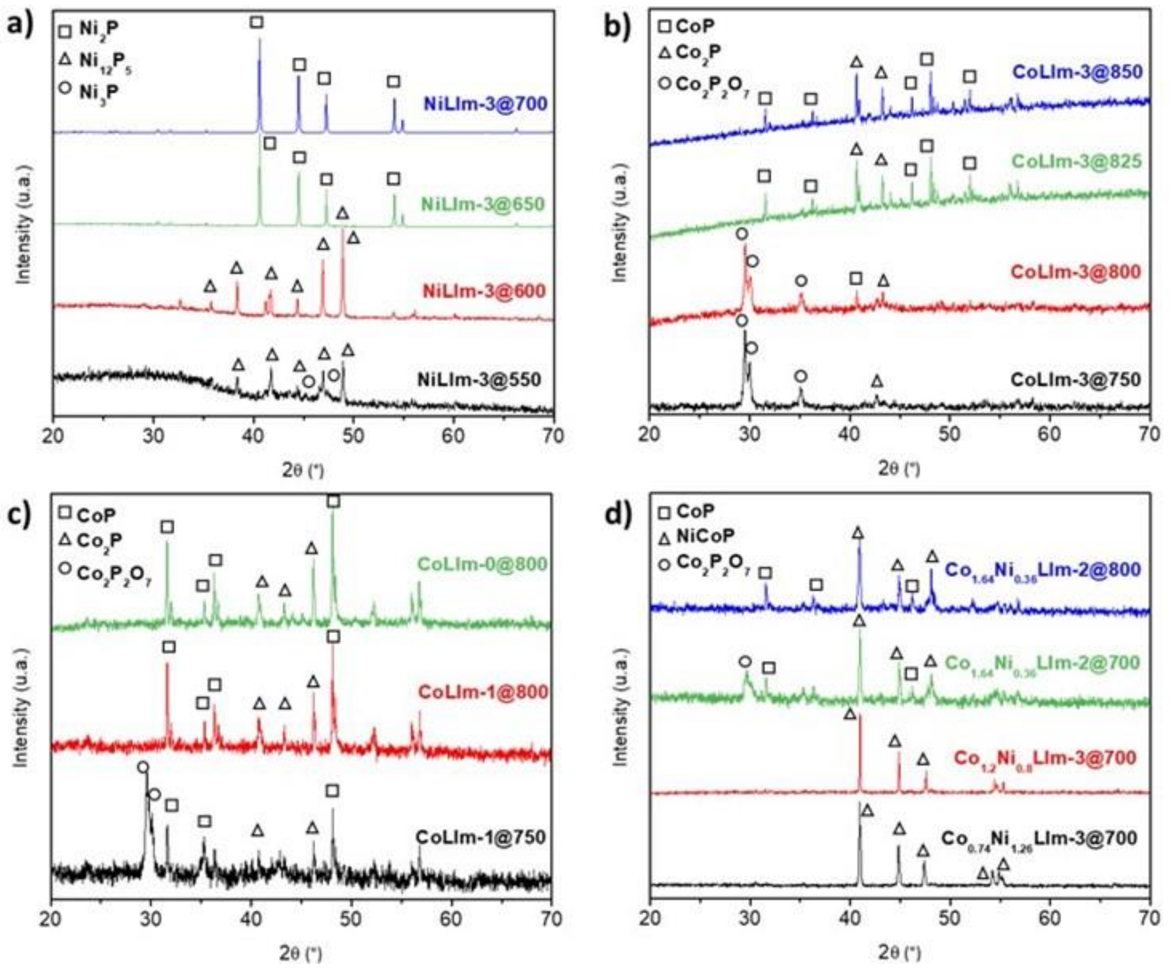

Figure S9. PXRD patterns of the resulting pyrolyzed derivatives from (a) **NiLIm-3**, (b) **CoLIm-3**, (c) **CoLIm-n** (n= 0, 1) and (d) **(Co$_x$Ni$_{2-x}$)LIm-n** (n=2, 3) at different temperatures under 5%H_2-Ar atmosphere (symbols indicate the reference patterns).

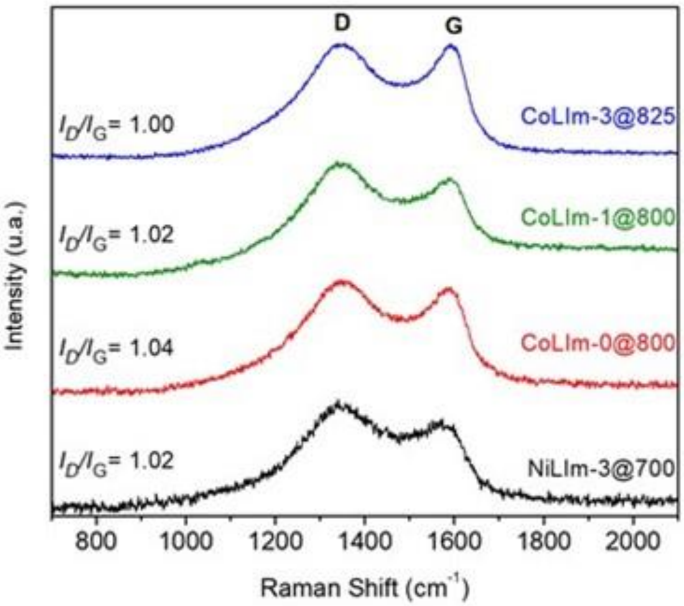

Figure S10. Raman spectra for selected metal phosphides.

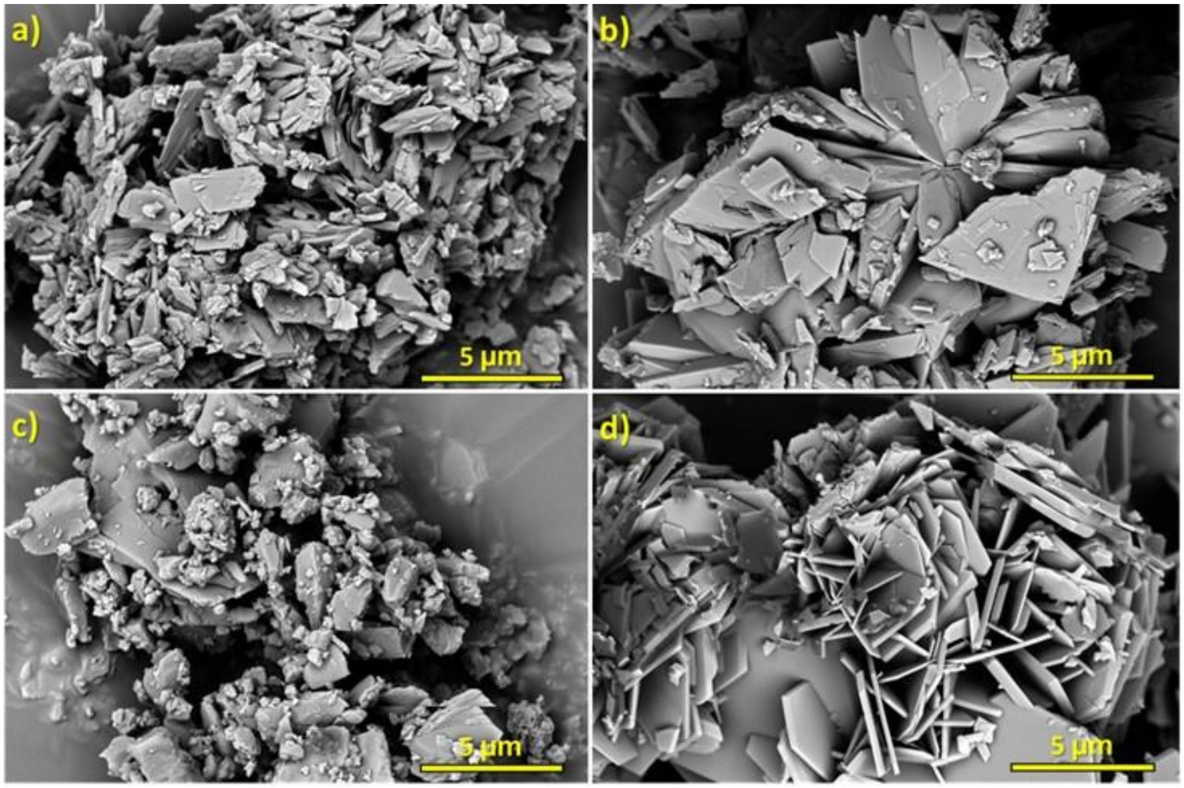

Figure S11. SEM images for precursors (a) **CoLIm-0**, (b) **CoLIm-1**, (c) **CoLIm-3** and (d) **NiLIm-3**.

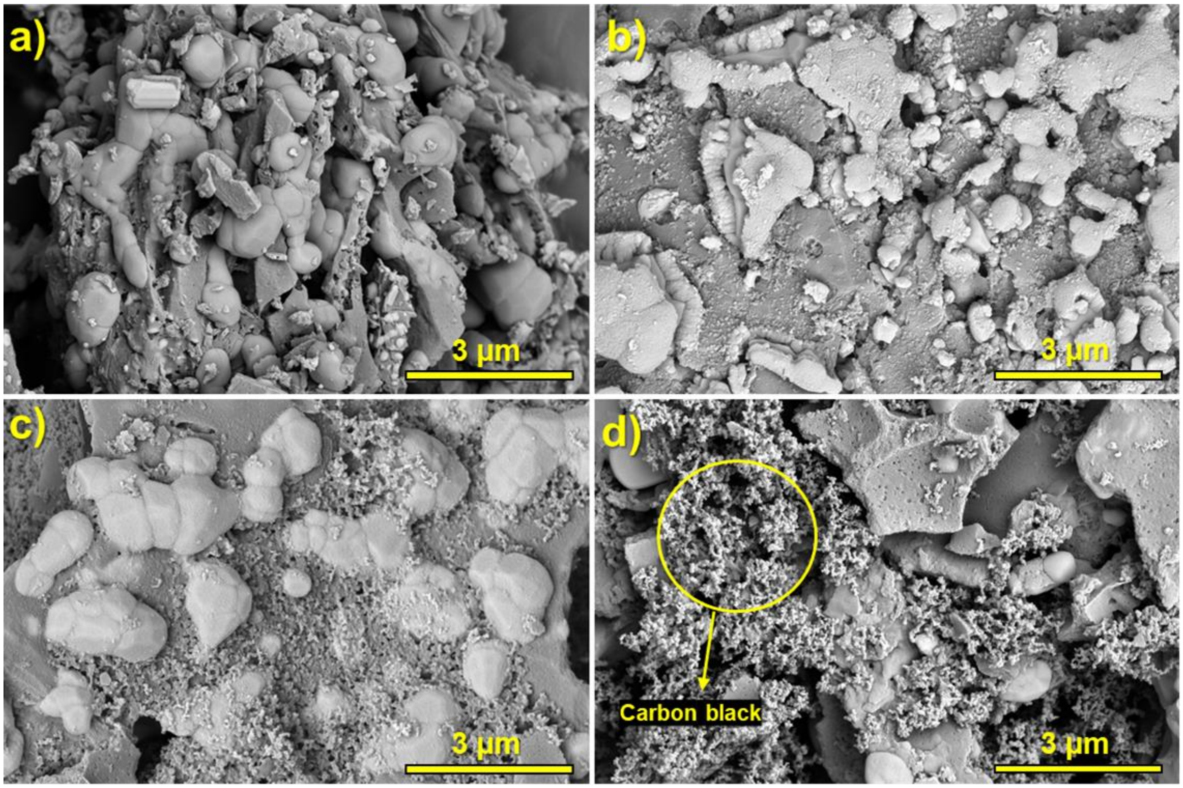

Figure S12. SEM images of (a) as-synthesized **CoLIm-0@800** and after conducting durability tests for (b) OER, (c) ORR and (d) HER.

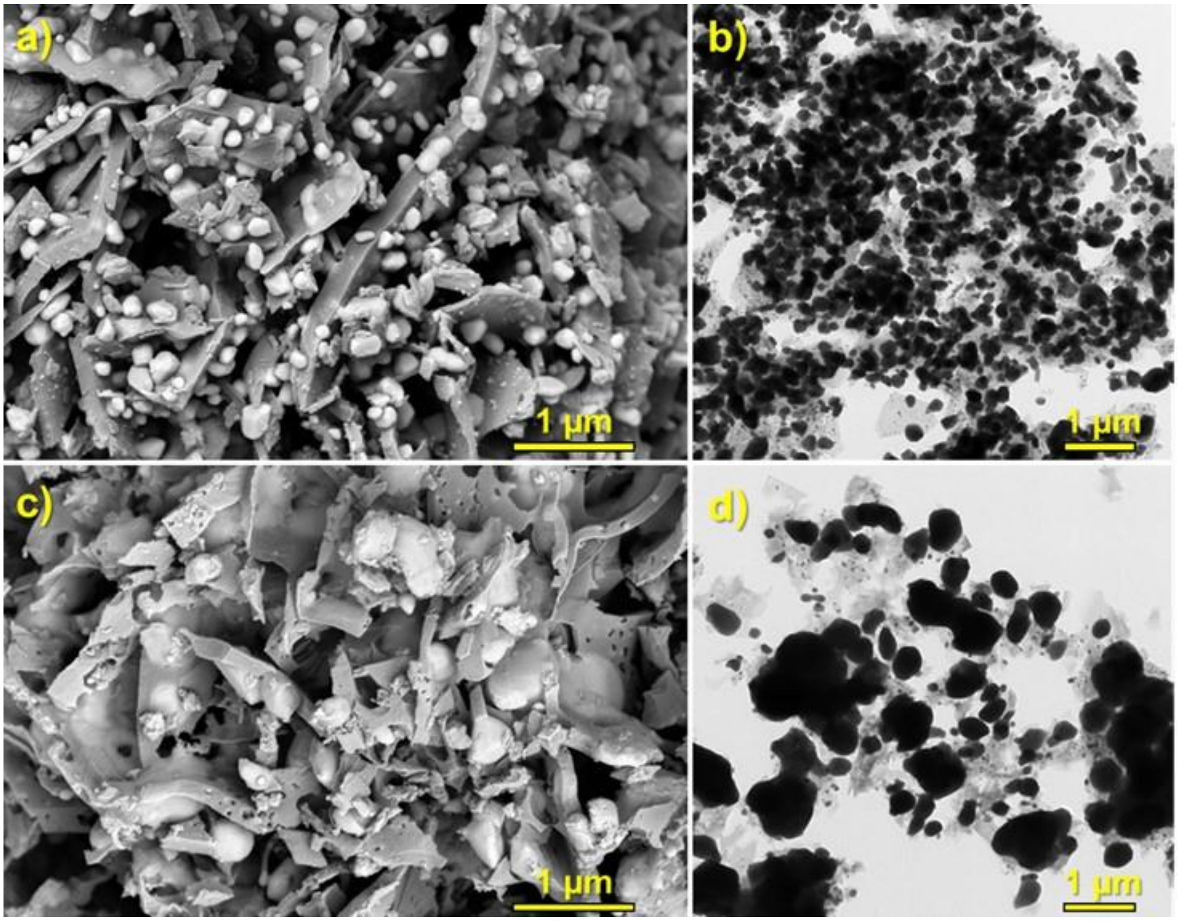

Figure S13. SEM and TEM images for (a,b) **NiLIm-3@700** and (c,d) **NiLIm-3@800**.

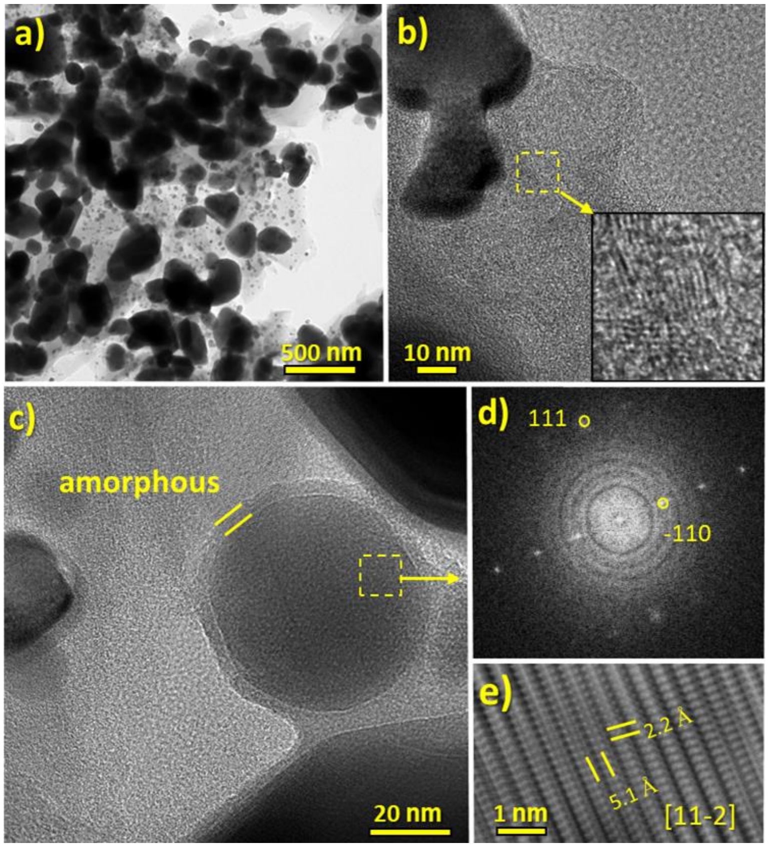

Figure S14. (a) TEM image of **NiLIm-3@700** and HRTEM image of (b) the graphitic carbon matrix and (c) **NiLIm-3@700** particle (figures d and e shows the indexation of the electron diffraction pattern corresponding to Ni_2P nanoparticles).

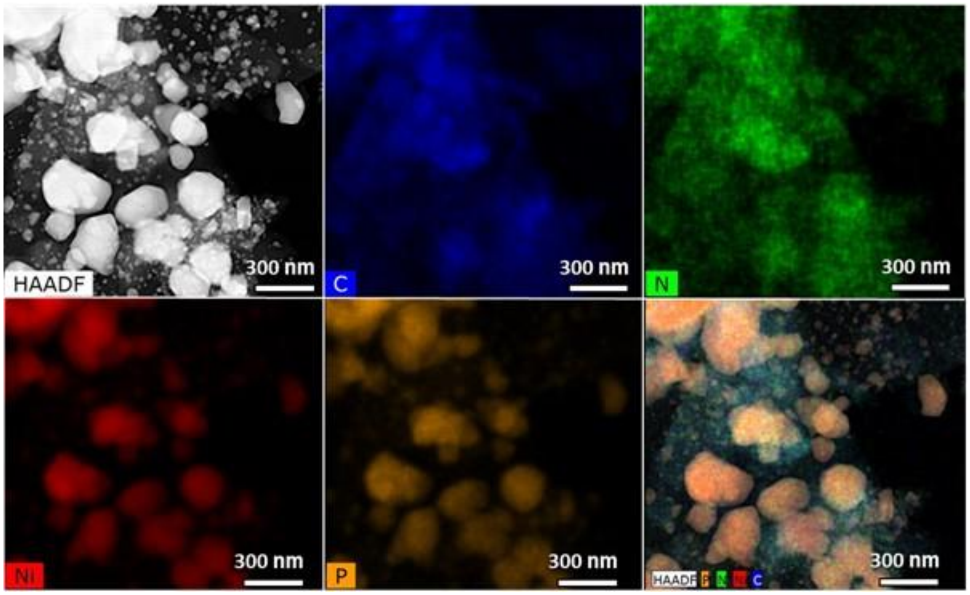

Figure S15. HAADF-EDX image and elemental distributions of C, N, Ni and P for **NiLIm-3@700.**

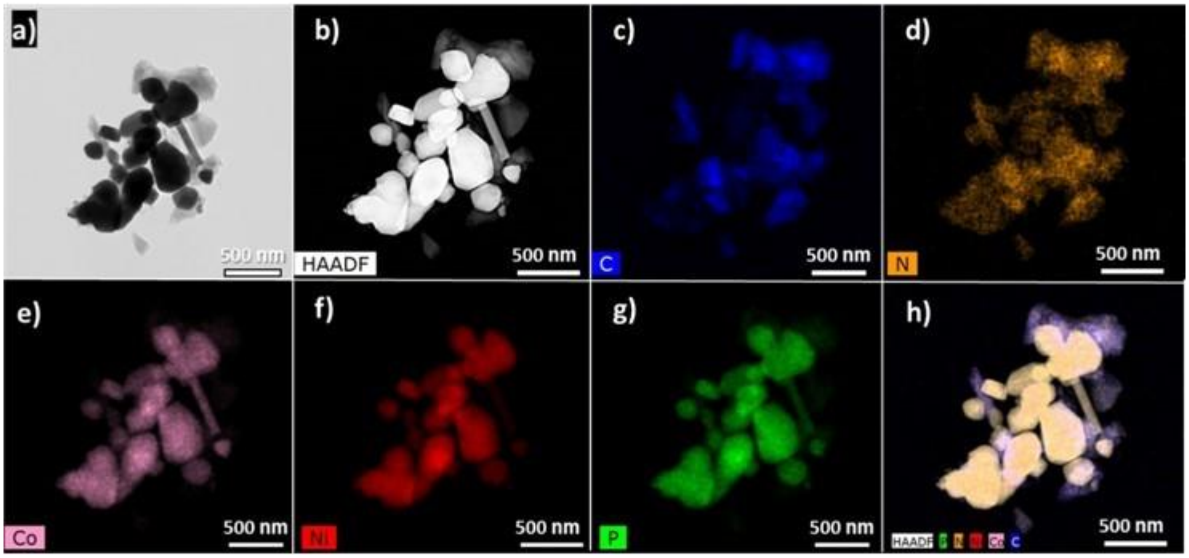

Figure S16. (a) TEM and (b) HAADF-EDX images with elemental distribution of (c) C, (d) N, (e) Co, (f) Ni, (g) P and (h) C+N+Co+Ni+P for **$Co_{0.74}Ni_{1.26}$LIm-3@700**.

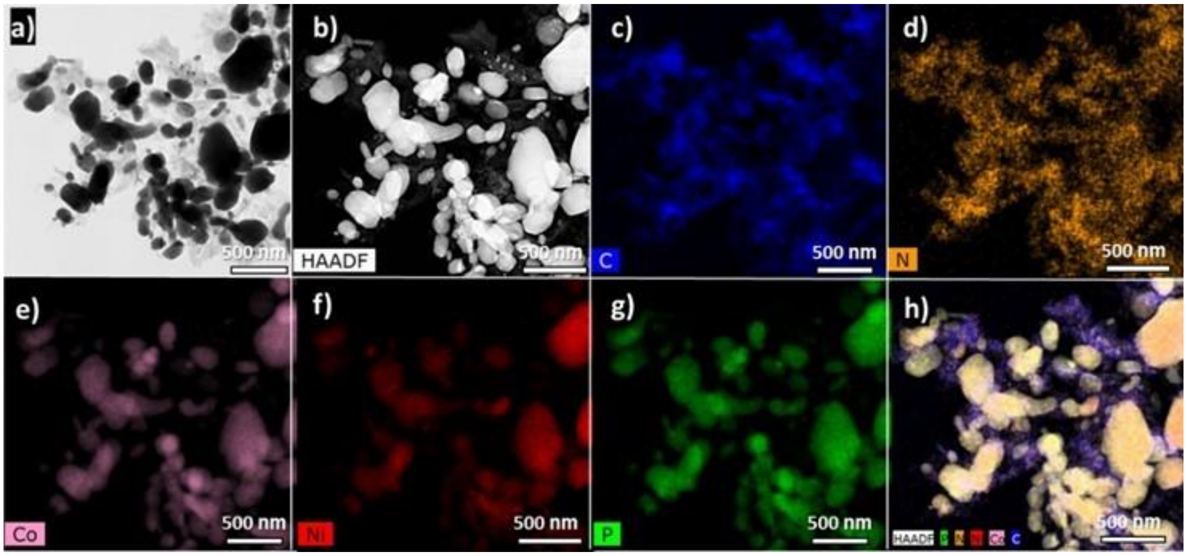

Figure S17. (a) TEM and (b) HAADF-EDX images with elemental distributions of (c) C, (d) N, (e) Co, (f) Ni, (g) P and (h) C+N+Co+Ni+P for **$Co_{1.64}Ni_{0.36}$LIm-2@800**.

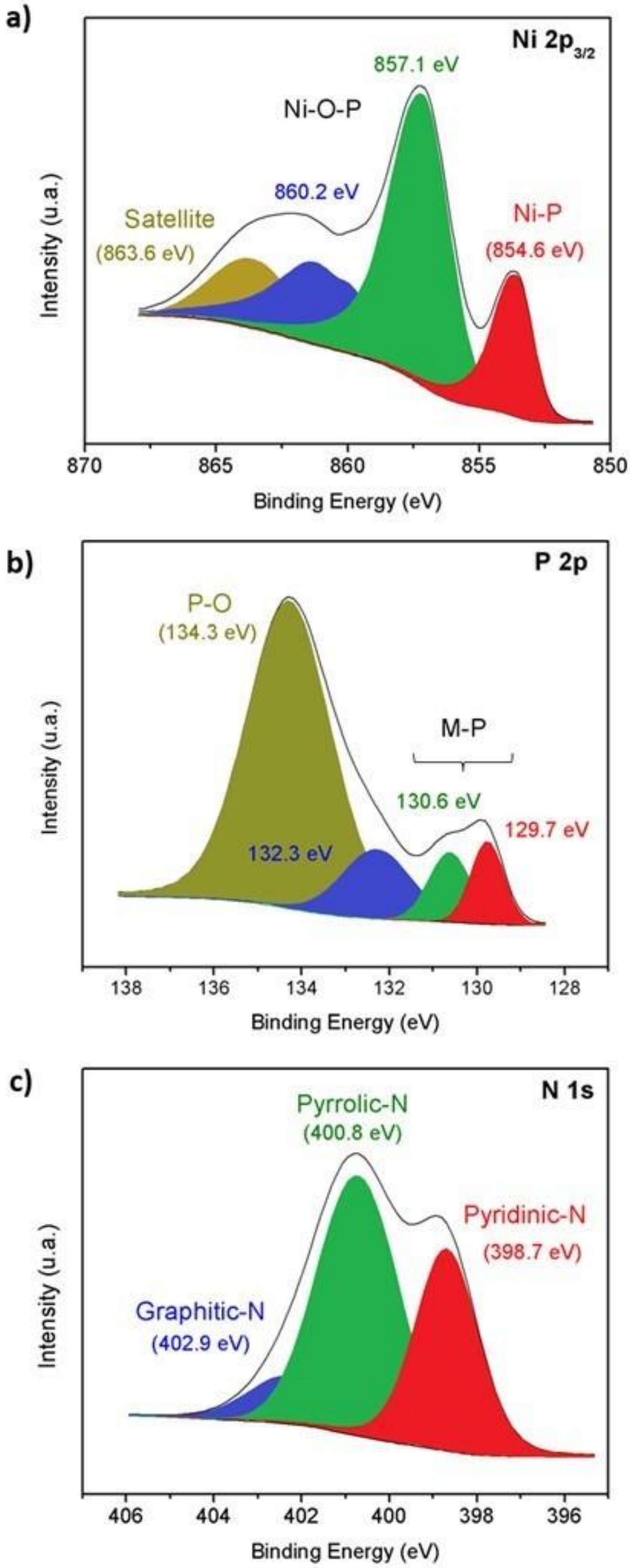

Figure S18. XPS spectra of (a) Ni $2p_{3/2}$, (b) P 2p and (c) N 1s regions for **NiLIm-3@700.**

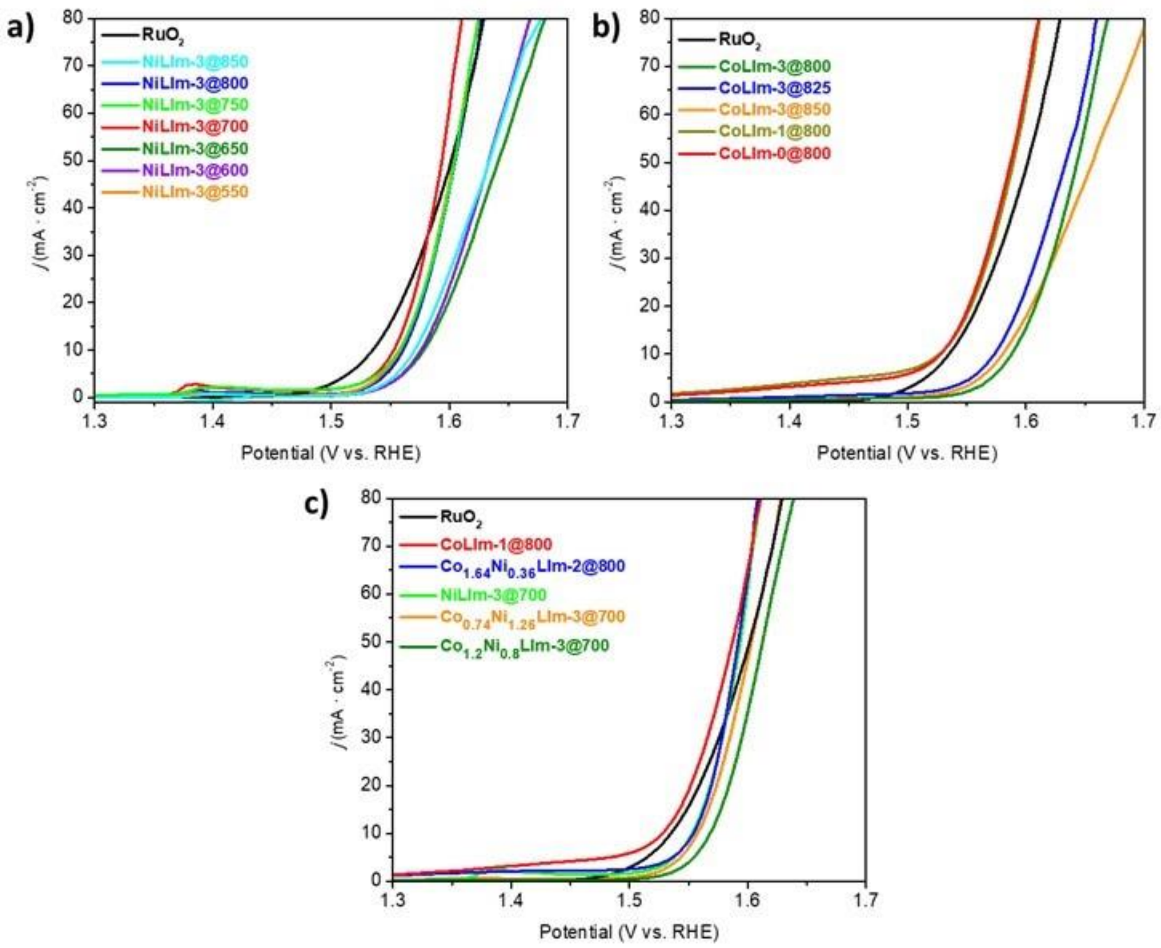

Figure S19. Oxygen evolution polarization curves of pyrolyzed materials derived from (a) **NiLIm-3**, (b) **CoLIm-n** (n= 0, 1, 3) and (c) **(Co_xNi_{2-x})LIm-n** (n=2, 3) compared with commercial RuO_2 in 1.0 M KOH.

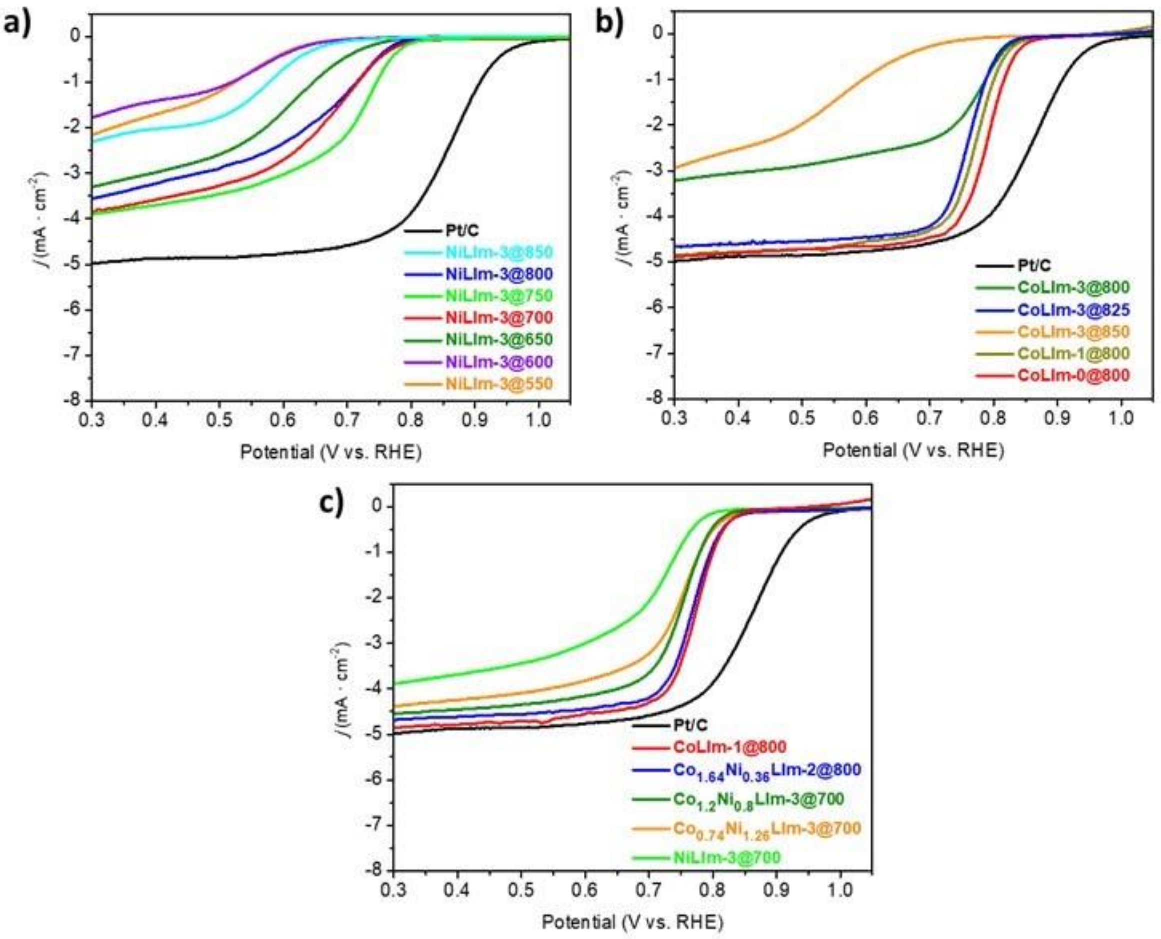

Figure S20. Oxygen reduction polarization curves of pyrolyzed materials derived from (a) **NiLIm-3**, (b) **CoLIm-n** (n= 0, 1, 3) and (c) **(Co_xNi_{2-x})LIm-n** (n=2, 3) compared with Pt/C electrocatalyst in 0.1 M KOH.

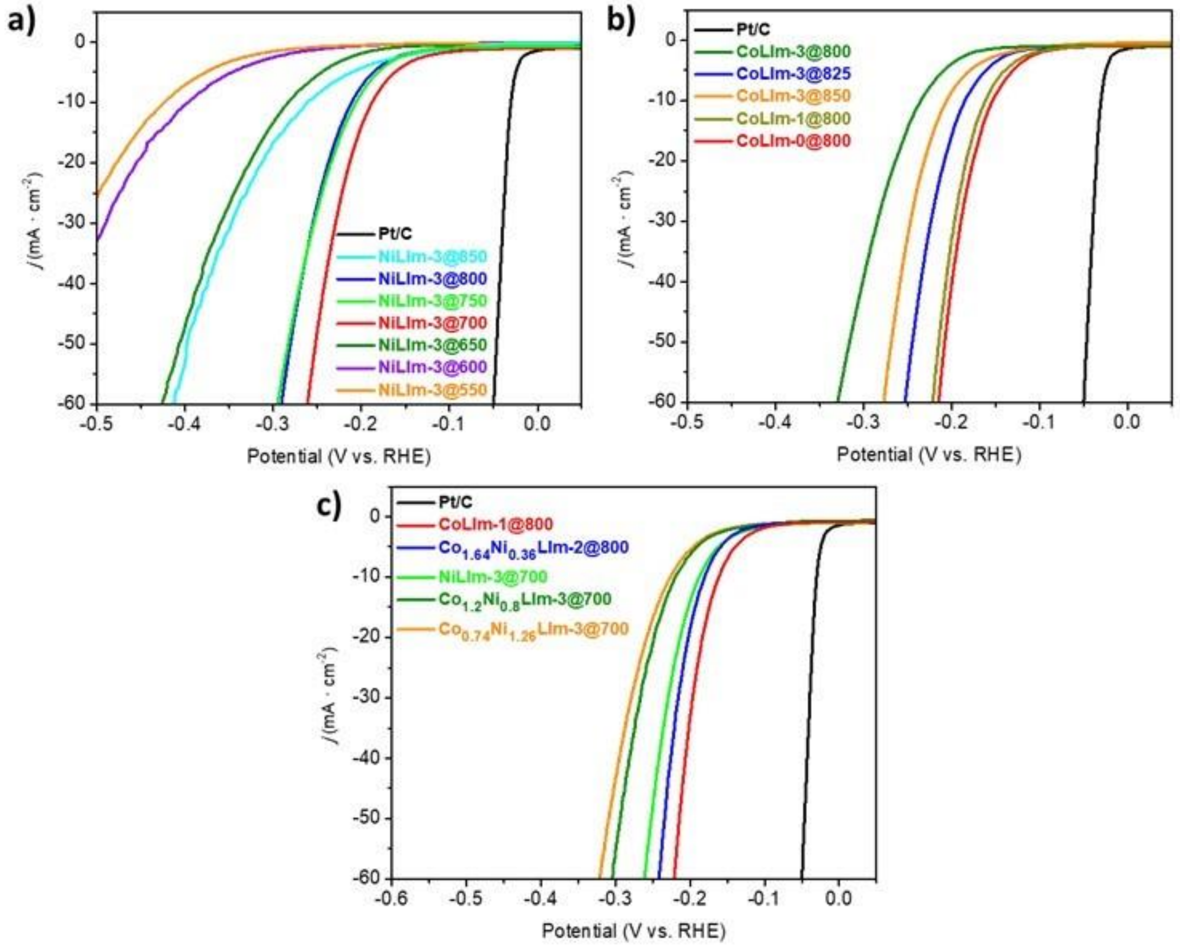

Figure S21. Hydrogen evolution polarization curves of pyrolyzed materials derived from (a) **NiLIm-3**, (b) **CoLIm-n** (n= 0, 1, 3) and (c) **(Co_xNi_{2-x})LIm-n** (n=2, 3) compared with Pt/C electrocatalyst in 0.5 M H_2SO_4.

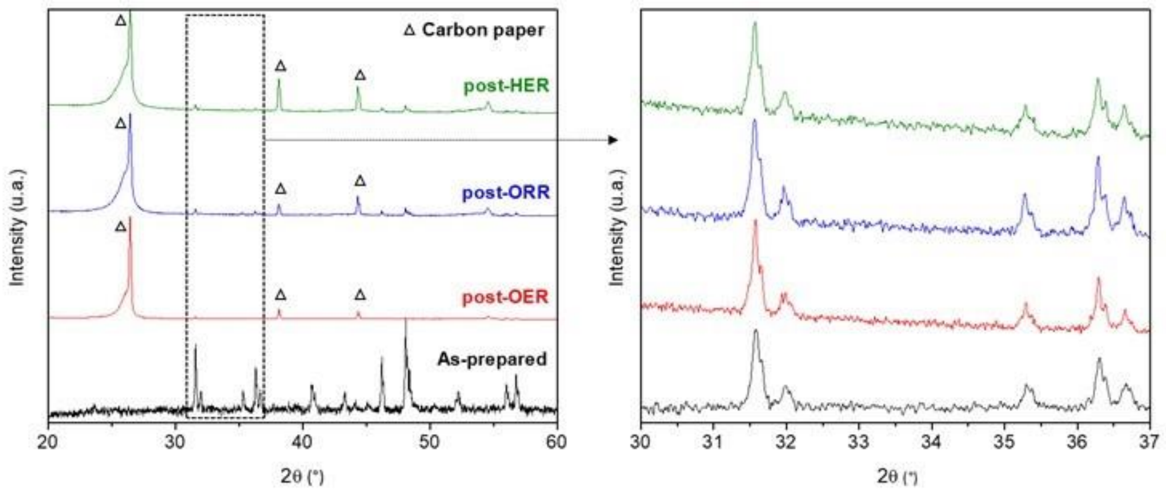

Figure S22. PXRD patterns after OER, ORR and HER tests of **CoLIm-0@800** compared to the as-prepared catalyst.

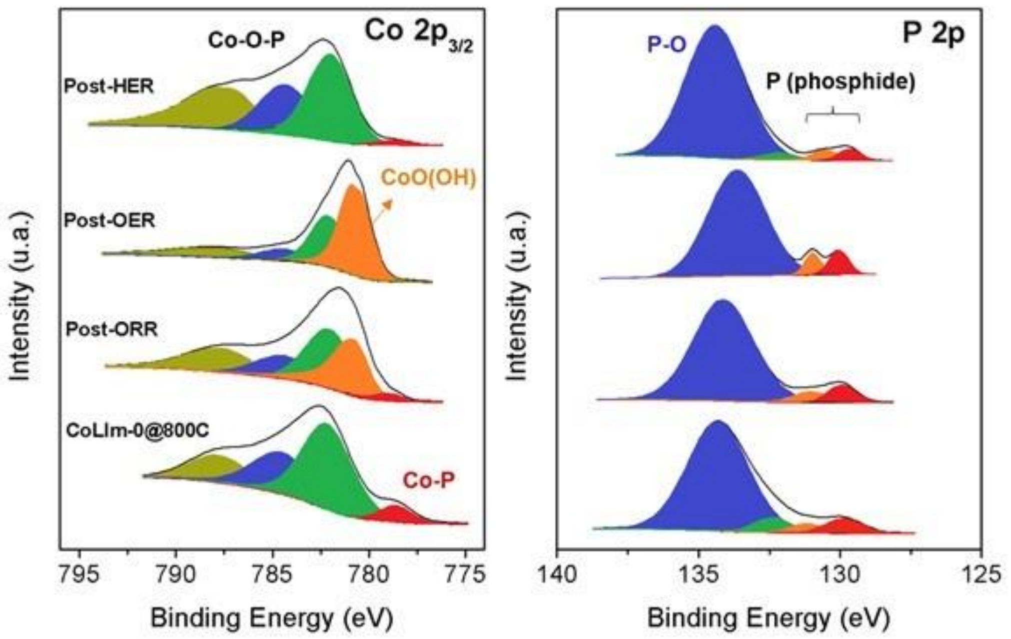

Figure S23. XPS spectra of Co $2p_{3/2}$ and P 2p regions after OER, ORR and HER tests of **CoLIm-0@800** compared to the as-prepared catalyst.

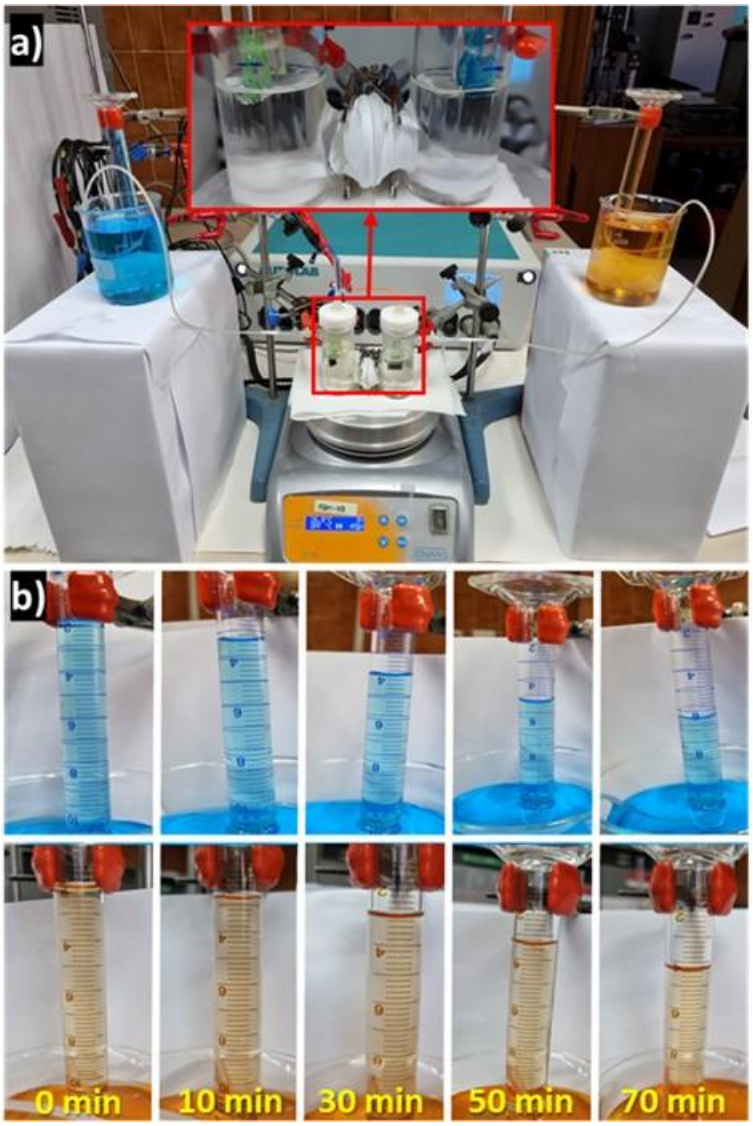

Figure S24. Photographs of (a) the homemade water splitting equipment and (b) gas production at different times (10 to 70 min. from left to right). Hydrogen is liberated at topside and oxygen at downside.

Table S1. Rietveld phase quantification and selected electrochemical properties for **CoLIm-0@800** after annealing in 5%H_2-Ar.

**5%H_2-Ar** ($mL·min^{-1}$)	**$Co_2P_2O_7$** (wt.%)	**Co_2P** (wt.%)	**CoP** (wt.%)	**η_{10} OER** (mV)	**$E_{onset}/E_{1/2}$ ORR** (V)	**η_{10} HER** (mV)
9	37	28	35	311	0.84/0.77	193
14	---	51	49	314	0.85/0.78	190
20	---	23	77	298	0.86/0.80	154
30	---	8	92	318	0.85/0.77	168

*η_{10}: overpotential value at 10 $mA·cm^{-2}$ (OER) or -10 $mA·cm^{-2}$ (HER); E_{onset} = onset potential; $E_{1/2}$ = half-wave potential.

Table S2. Elemental analysis for **MLIm-n** (n=0, 1, 3) and **(Co_xNi_{2-x})LIm-n** (n=2, 3) compounds**.**

**Sample label**	**C(%)**		**H(%)**		**N(%)**		**Co(%)**		**Ni(%)**	
	**Found**	**Calc.**	**Found**	**Calc.**	**Found**	**Calc.**	**Found**	**Calc.**	**Found**	**Calc.**
**CoLIm-3**	22.94	22.84	4.00	3.81	15.01	14.54	-	-	-	-
**CoLIm-1**	23.73	24.36	3.43	3.35	15.32	15.50	-	-	-	-
**CoLIm-0**	25.46	25.19	3.42	3.08	16.28	16.04	-	-	-	-
**NiLIm-3**	22.77	22.86	3.96	3.81	14.97	14.55	-	-	-	-
**ZnLIm-2**	16.61	16.88	3.27	3.38	7.97	7.88	-	-	-	-
**$Co_{0.74}Ni_{1.26}$LIm-3**	22.77	22.89	3.30	3.84	14.66	14.57	7.24	7.56	12.33	12.66
**$Co_{1.2}Ni_{0.8}$LIm-3**	22.77	22.87	3.30	3.84	14.66	14.56	12.41	12.25	7.85	8.03
**$Co_{1.64}Ni_{0.36}$LIm-2**	23.49	23.59	2.74	3.60	15.05	15.01	17.21	17.27	3.72	3.73

Table S3. Crystallographic data for **(Co_xNi_{2-x})LIm-n** (n=2, 3) compounds obtained by Le Bail fit.

**Phase**	**$Co_{0.74}Ni_{1.26}$LIm-3**	**$Co_{1.2}Ni_{0.8}$LIm-3**	**$Co_{1.64}Ni_{0.36}$LIm-2**
**Space group**	P-1	P-1	P-1
**Chemical formula**	$Co_{0.74}Ni_{1.26}P_2C_{11}H_{22}N_6O_{10}$	$Co_{1.2}Ni_{0.8}P_2C_{11}H_{22}N_6O_{10}$	$Co_{1.64}Ni_{0.36}P_2C_{11}H_{20}N_6O_9$
**a (Å)**	10.4414(8)	10.4405(10)	11.5751(12)
**b (Å)**	11.2846(10)	11.3457(10)	9.7294(11)
**c (Å)**	10.7007(7)	10.7192(9)	8.9383(10)
**α (°)**	83.641(6)	83.551(7)	108.565(9)
**β (°)**	79.494(4)	79.364(5)	92.529(9)
**γ (°)**	55.534(3)	55.503(3)	73.718(8)
**V ($Å^3$)**	1022.04(13)	1028.45(14)	914.84(21)
**R_{wp} (%)**	4.12	3.42	3.13

Table S4. ATR-FTIR bands assignments for **CoIIm-1**.

**Band** (cm^{-1})	**Assignments** [1-4]
3350-3000 (br, m)	$\nu 3 + \nu 1$ stretches of H-bonded H_2O, C-OH (ETID)
3165 (w)	C-H stretch (Im ring)
3130 (w)	CH_3 stretch (ETID)
3062 (w)	N-H, C-H (Im ring)
2951 (w), 2857 (w), 2694 (w)	C-H (Im ring)
1657 (w)	Im ring I band
1542 (m)	C=N (Im ring)
1491 (m)	C=C, N=C-N (Im ring)
1440 (m), 1326 (m)	ν (Im ring)
1264 (m), 1179 (m)	PO_3 antisymmetric stretch
1246 (m)	δ (CH)
1146 (m)	ν (Im ring)
1117 (m), 1070 (s), 1050 (s)	P=O, P-O, PO_3 vibrations
1099 (m)	δ (C–H)
1028 (s)	δ (C–H)
985 (s), 848 (m), 769 (s)	P-C stretch
938 (s), 893 (m), 824 (s)	δ (Im ring), γ (C–H)
911 (m)	P-O stretch
737 (s)	γ C-H (Im ring)
665 (sh)	γ C-H (Im ring)
656 (s), 630 (m)	γ (Im ring)
616 (s)	γ (Im ring)
587 (m), 567 (s), 552 (sh)	PO_3 band
534 (m), 517 (s), 507 (s)	Co-N, Co-O

*Notations: br, broad; s, strong; m, medium; w, weak; sh, shoulder; ETID, etidronate; Im, imidazole.

Table S5. H-bond distances for **ZnLIm-2**.

D-H···A	D-H (Å)	H···A (Å)	D···A (Å)	D-H···A (Å)
O(4)-H(4)···O(14)	0.84	1.92	2.754(3)	175.9
O(7)-H(7)···O(13)	0.84	1.72	2.540(3)	164.6
O(10)-H(10)···O(3)	0.84	1.66	2.428(3)	150.8
O(11)-H(11)···O(5)	0.84	2.02	2.862(3)	174.9
O(15)-H(15A)···O(6)	0.89	1.85	2.707(3)	161.3
O(15)-H(15B)···O(13)	0.89	2.08	2.696(3)	125.5
O(16)-H(16B)···O(12)	0.88	2.04	2.876(3)	158.6
N(3)-H(3)···O(13)	0.88	1.91	2.747(4)	157.2
N(4)-H(4D)···O(8)	0.88	1.92	2.781(4)	164.3
N(1)-H(1)···O(9)	0.88	1.90	2.767(4)	166.5
N(2)-H(2)···O(1)	0.88	1.94	2.812(4)	172.6

Table S6. H-bond distances for **NiLIm-3**.

D-H···A	D-H (Å)	H···A (Å)	D···A (Å)	D-H···A (Å)
N(2)-H(2)···O(2)	0.88	1.95	2.777(3)	156.7
N(4)-H(4)···O(5)	0.88	1.96	2.789(3)	157.2
O(7)-H(7)···O(4)	0.789(17)	1.960(18)	2.726(2)	164(3)
O(8)-H(8A)···O(10)	0.87	1.90	2.707(2)	152.0
O(8)-H(8B)···O(4)	0.87	1.98	2.825(2)	163.0
O(9)-H(9A)···O(10)	0.74(4)	2.19(4)	2.834(2)	147(4)
O(9)-H(9B)···O(1)	0.87(4)	1.81(4)	2.644(2)	162(3)
O(10)-H(10A)···O(2)	0.87	1.83	2.695(2)	178.9

Table S7. H-bond distances for **CoLIm-3**.

**D-H···A**	**D-H (Å)**	**H···A (Å)**	**D···A (Å)**	**D-H···A (Å)**
O(9)-H(9A)···O(10)	0.89	1.89	2.717(2)	154.5
O(9)-H(9B)···O(3)	0.89	1.93	2.810(2)	170.3
O(8)-H(8A)···O(10)	0.88	2.03	2.826(2)	150.4
O(8)-H(8B)···O(7)	0.88	2.11	2.682(2)	122.2
O(10)-H(10E)···O(6)	0.87	1.84	2.700(2)	171.1
O(4)-H(4)···O(3)	0.856(10)	1.932(13)	2.748(2)	159(2)
N(4)-H(4A)···O(1)	0.88	1.98	2.810(2)	157.9
N(2)-H(2)···O(6)	0.88	1.95	2.784(3)	157.6
N(6A)-H(6A)···O(6)	0.88	2.03	2.888(5)	165.0

Table S8. Elemental analysis for metal phosphides derived from **NiLIm-3**.

**Sample Label**	**C(%)**	**H(%)**	**N(%)**
**NiLIm-3@550**	12.576	0.589	5.268
**NiLIm-3@600**	12.263	0.286	4.898
**NiLIm-3@650**	13.377	0.408	4.733
**NiLIm-3@700**	16.062	0.723	2.461
**NiLIm-3@750**	12.592	0.686	1.577
**NiLIm-3@800**	3.599	0.091	0.340
**NiLIm-3@850**	0.296	0.000	0.040

Table S9. Comparison of OER, HER and ORR performance of selected electrocatalysts with recent studies under similar electrochemical measurement conditions.

**Electrocatalyst** (*Crystalline phases*)	**Organic precursors**	**OER**		**HER**		**ORR**		**References**
		**η_{10}** (mV)	**TS** ($mV \cdot dec^{-1}$)	**η_{10}** (mV)	**TS** ($mV \cdot dec^{-1}$)	**E_{onset}** (V)	**$E_{1/2}$** (V)	
**CoLIm-0@800*** (80% *CoP + 20% Co_2P*)	**[1]HEDP + Imidazole**	**298**	**64.9**	**156**	**79.7**	**0.86**	**0.80**	**This work**
**NiLIm-3@700*** (*Ni_2P*)	**[1]HEDP + Imidazole**	**321**	**55.2**	**185**	**88.2**	**0.78**	**0.66**	**This work**
FeNiP/NPCS* (*Fe_2P, Ni_2P*)	[1]HEDP + [2]PVP + (g-C_3N_4)	318	95	126	64	N/A	0.84	5
Fe-Co_2P@Fe-N-C*(*Fe-doped Co_2P*)	[1]HEDP + Melamine	300	79	-	-	0.92	0.88	6
CoP@rGO*(*CoP*)	[3]PA	280	75	-	-	-	-	7
Co-P@PC* (*CoP + Co_2P*)	[1]HEDP	280	53	72	49	-	-	8
CoP@SNC* (*CoP*)	[1]HEDP + Thiourea	350	68	-	-	0.87	0.79	9
Co_2P/NPC-900 (*Co_2P*)	GO + DMF + $Cl_6N_3P_3$	320	66.6	-	-	0.94	0.81	10
CoP/NCNHP (*CoP*)	[4]MeIm	310	70	140	53	-	-	11
CoP NFs (*CoP*)	$Co_3[Co(CN)_6]_2$	323	49.6	122	54.8	-	-	12
Co-P@NC-800 (*CoP + Co_2P*)	[5]Bpy + [2]PVP + [6]TCPP + DMF	370	79	-	-	-	-	13
Co-P/NC (*CoP + Co_2P*)	[4]MeIm	319	52	-	-	-	-	14
Fe-NiCoP@C (*NiCoP*)	Glucose solution	270	36	-	-	0.81	N/A	15
CoP_x@CNS (*CoP + Co_2P*)	[4]MeIM	286	70	-	-	0.83	0.76	16
FeCo/FeCoP@NP-CF* (*Fe_2P + Co_2P + FeCo*)	[7]PAM + Melamine	-	-	-	-	N/A	0.85	17
CoP NCs (*CoP*)	Urea	-	-	-	-	0.80	0.70	18
Co_2P@CoNPG-800 (*Co_2P*)	GO + Melamine	-	-	-	-	0.87	0.80	19
CoP-PBSCF (*CoP*)	[2]PVP + DMF	-	-	-	-	N/A	0.75	20
CoMoP-C* (*CoMoP*)	[8]PMIDA	-	-	155	72.3	-	-	21
CoP/Co_2P@NC* (*CoP + Co_2P*)	[9]EDTMP	-	-	126	79	-	-	22
CoP/Ni_2P@NC* (*CoP + Ni_2P*)	[9]EDTMP	-	-	91	62	-	-	23
Co_2P@NPG* (*Co_2P*)	[10]PA + Acrylamide	-	-	103	58	-	-	24
CoP@N,P–C* (*CoP*)	[1]HEDP + [2]PVP	-	-	140	63	-	-	25
NiP@N,P–C* (*Ni_2P*)	[1]HEDP + [2]PVP	-	-	196	79	-	-	25

*Derived from metal phosphonates.

Abbreviations: [1]HEDP = 1-Hydroxyethylidene-1,1-diphosphonic acid; [2]PVP = poly(vinyl pyrrolidone); [3]PA = myo-inositol 1,2,3,4,5,6-hexakisphosphate; [4]MeIm = 2-Methylimidazole; [5]Bpy = 4,4′-Bipyridine; [6]TCPP = 4,4′,4″,4‴-((4,4′,4″,4‴-(porphyrin-5,10,15,20-tetrayl)tetrakis(benzoyl))tetrakis-(azanediyl))tetrabenzoic acid;; [7]PAM = polyacrylamide; [8]PMIDA = Phosphonomethyliminodiacetic acid; [9]EDTMP = ethylenediamine tetra(methylene phosphonic acid); [10]PA = Phytic acid.

Electrochemical parameters: η_{10} = overpotential value at 10 $mA \cdot cm^{-2}$ (OER) or -10 $mA \cdot cm^{-2}$ (HER); TS = tafel slope; E_{onset} = onset potential; $E_{1/2}$ = half-wave potential.

Table S10. Summary of the crystalline phases and electrochemical properties for selected electrocatalysts.

Sample label	Crystalline phases	OER	ORR		HER
		η_{10} (mV)	E_{onset} (V)	$E_{1/2}$ (V)	η_{10} (mV)
**NiLIm-3@550**	$Ni_{12}P_5 + Ni_3P$	328	0.66	0.51	424
**NiLIm-3@600**	$Ni_{12}P_5 + Ni_3P$	345	0.66	0.55	399
**NiLIm-3@650**	Ni_2P^a (96%) + $Ni_{12}P_5$ (4%)	347	0.77	0.60	284
**NiLIm-3@700**	Ni_2P^a	321	0.78	0.66	185
**NiLIm-3@750**	Ni_2P^a	329	0.80	0.71	202
**NiLIm-3@800**	Ni_2P^a	331	0.78	0.65	211
**NiLIm-3@850**	Ni_2P^a	341	0.69	0.57	263
**CoLIm-3@800**	$Co_2P_2O_7 + Co_xP$	359	0.84	0.76	236
**CoLIm-3@850**	Co_2P^b (52%) + CoP (42%)	349	0.73	0.55	208
**$Co_{0.74}Ni_{1.26}$LIm-3@750**	$CoNiP^a$ (97%) + CoP (3%)	328	0.84	0.74	225
**$Co_{1.2}Ni_{0.8}$LIm-3@750**	$CoNiP^a$ (95%) + CoP (5%)	338	0.84	0.74	219
**$Co_{1.64}Ni_{0.36}$LIm-2@800**	$CoNiP^a$ (60%) + CoP (40%)	324	0.85	0.77	180
**$Co_{1.4}Ni_{0.6}$L@800**	$CoNiP^a$ (96%) + CoP (4%)	331	0.77	0.61	208

[a]hexagonal phase; [b]orthorhombic phase; η_{10} = overpotential value at 10 mA·cm^{-2} (OER) or -10 mA·cm^{-2} (HER); E_{onset} = onset potential; $E_{1/2}$ = half-wave potential.

References

[1] Komova, O.V.; Mukha, S.A.; Ozerova, A.M.; Bulavchenko, O.A.; Pochtar, A.A.; Ishchenko, A.V.; Odegova, G.V.; Suknev, A.P.; Netskina, O.V. New Solvent-Free Melting-Assisted Preparation of Energetic Compound of Nickel with Imidazole for Combustion Synthesis of Ni-Based Materials. *Nanomaterials* **2021**, *11*, 3332.

[2] Mesu, J.G.; Visser, T.; Soulimani, F.; Weckhuysen, B.M. Infrared and Raman spectroscopic study of pH-induced structural changes of L-histidine in aqueous environment. *Vib. Spectrosc.* **2005**, *39*, 114–125.

[3] Wu, D.; Kang, R.; Guo, J.; Liu, Z.; Wan, C.; Jin, Z. On the reaction mechanism of a hydroxyethylidene diphosphonic acid-based electrolyte for electrochemical mechanical polishing of copper. *Electrochem. Commun.* **2019**, *103*, 48–54.

[4] Xia, Y.; Mao, Z.; Jin, F.; Guan, Y.; Zheng, A. Synthesis of 1-hydroxy ethylidene-1,1-diphosphonic ammonium and the promise of this ammonium salt as an intumescent flame retardant in polystyrene. *Polym. Degrad. Stab.* **2014**, *102*, 186-194.

[5] Jin-Tao Ren; Yan-Sun Wang; Lei Chen; Li-Jiao Gao; Wen-Wen Tian; Zong-Yong Yuan. Binary FeNi phosphides dispersed on N, P-doped carbon nanosheets for highly efficient overall water splitting and rechargeable Zn-air batteries. *Chem. Eng. J.* **2020**, *389*, 124408.

[6] Xian-Wei Lv; Wei-Shan Xu; Wen-Wen Tian; Hao-Yu Wang; Zhong-Yong Yuan. Activity Promotion of Core and Shell in Multifunctional Core–Shell Co_2P@NC Electrocatalyst by Secondary Metal Doping for Water Electrolysis and Zn-Air Batteries. *Small* **2021**, *17*, 2101856.

[7] Gong Zhang; Guichang Wang; Yang Liu; Huijuan Liu; Jiuhui Qu; Jinghong Li. Highly Active and Stable Catalysts of Phytic Acid-Derivative Transition Metal Phosphides for Full Water Splitting. *J. Am. Chem. Soc.* **2016**, *138*, 14686−14693.

[8] Jiadong Wu; Depeng Wang; Shuao Wan; Huiling Liu; Cheng Wang; Xun Wang. An Efficient Cobalt Phosphide Electrocatalyst Derived from Cobalt Phosphonate Complex for All-pH Hydrogen Evolution Reaction and Overall Water Splitting in Alkaline Solution. *Small* **2020**, *16*, 1900550.

[9] Tao Meng; Yi-Ning Hao; Lirong Zheng; Minhua Cao. Organophosphoric acid-derived CoP quantum dots@S,N-codoped graphite carbon as a trifunctional electrocatalyst for overall water splitting and Zn–air batteries. *Nanoscale* **2018**, *10*, 14613-14626.

[10] Qi Shao; Tianjiao Li; Heng-guo Wang; Yanhui Li; Zhenjun Si; Qian Duan. A facile one-pot synthesis of Co_2P nanoparticle-encapsulated doped carbon nanotubes as bifunctional electrocatalysts for high-performance rechargeable Zn–air batteries. *ACS Sustain. Chem. Eng.* **2020**, *8*, 6422-6432.

[11] Yuan Pan; Kaian Sun; Shoujie Liu; Xing Cao; Konglin Wu; Weng-Chon Cheong; Zheng Chen; Yu Wang; Yang Li; Yunqi Liu; Dingsheng Wang; Qing Peng; Chen Chen; Yadong Li. Core-Shell ZIF-8@ZIF

Derived CoP Nanoparticle-Embedded N-Doped Carbon Nanotube Hollow Polyhedron for Efficient Overall Water Splitting. *J. Am. Chem. Soc.* **2018**, *140*, 2610-2618.

[12] Lvlv Ji; Jianying Wang; Xue Teng; Thomas J. Meyer; Zuofeng Chen. CoP Nanoframes as Bifunctional Electrocatalysts for Efficient Overall Water Splitting. *ACS Catal.* **2020**, *10*, 1, 412–419.

[13] Mengke Zhai; Fei Wang; Hongbin Du. Transition-Metal Phosphide–Carbon Nanosheet Composites Derived from Two-Dimensional Metal-Organic Frameworks for Highly Efficient Electrocatalytic Water-Splitting. *ACS Appl. Mater. Interfaces* **2017**, *9*, 46, 40171–40179.

[14] Bo You; Nan Jiang; Meili Sheng; Sheraz Gul; Junko Yano; Yujie Sun. High-Performance Overall Water Splitting Electrocatalysts Derived from Cobalt-Based Metal−Organic Frameworks. *Chem. Mater.* **2015**, *27*, 22, 7636-7642.

[15] Yao Kanga; Shuo Wanga; Siqi Zhua; Haixing Gao; Kwan San Hui; Cheng-ZongYuan; Hong Yin; Feng Bin; Xi-Lin Wu; Wenjie Mai; Ling Zhu; Maocong Hu; Feng Liang; Fuming Chen; Kwun Nam Hui. Iron-modulated nickel cobalt phosphide embedded in carbon to boost power density of hybrid sodium–air battery. *Applied Catalysis B: Environmental* **2021**, *285*, 119786.

[16] Chun-Chao Hou; Lianli Zou; Yu Wang; Qiang Xu. MOF-Mediated Fabrication of a Porous 3D Superstructure of Carbon Nanosheets Decorated with Ultrafine Cobalt Phosphide Nanoparticles for Efficient Electrocatalysis and Zinc–Air Batteries. *Angew. Chem. Int. Ed.* **2020**, *59*, 21360-21366.

[17] Hua-Jie Niu; Shi-Yi Lin; Yu-Ping Chen; Jiu-Ju Feng; Qian-Li Zhang; Ai-Jun Wang.Hydrogel derived FeCo/FeCoP embedded in N, P-codoped 3D porous carbon framework as a highly efficient electrocatalyst for oxygen reduction reaction. *Applied Surface Science* **2021**, *536*, 147950.

[18] Hongchao Yang; Yejun Zhang; Feng Hu; Qiangbin Wang. Urchin-like CoP Nanocrystals as Hydrogen Evolution Reaction and Oxygen Reduction Reaction Dual-Electrocatalyst with Superior Stability. *Nano Lett.* **2015**, *15*, 7616-7620.

[19] Hao Jiang; Chang Li; Haibo Shen; Yisi Liu; Wenzhang Li; Jie Li.Supramolecular gel-assisted synthesis Co_2P particles anchored in multielement co-doped graphene as efficient bifunctional electrocatalysts for oxygen reduction and evolution. *Electrochim. Acta* **2017**, *231*, 344-353.

[20] Ya-Qian Zhang; Hong-Biao Tao; Zhou Chen; Meng Li; Yi-Fei Sun; Bin Hua; Jing-Li Luo. In situ grown cobalt phosphide (CoP) on perovskite nanofibers as an optimized trifunctional electrocatalyst for Zn–air batteries and overall water splitting. *J. Mater. Chem. A* **2019**, *7*, 26607-26617.

[21] Sayed M. El-Refaei; Patrícia A. Russo; Patrick Amsalem; Nobert Koch; Nicola Pinna. The Importance of Ligand Selection on the Formation of Metal Phosphonate-Derived CoMoP and $CoMoP_2$ Nanoparticles for Catalytic Hydrogen Evolution. *ACS Appl. Nano Mater.* **2020**, *3*, 5, 4147-4156.

[22] Xianwei Lv; Jintao Ren; Yansu Wang; Yuping Liu; Zhong-Yong Yuan. Well-Defined Phase-Controlled Cobalt Phosphide Nanoparticles Encapsulated in Nitrogen-Doped Graphitized Carbon

Shell with Enhanced Electrocatalytic Activity for Hydrogen Evolution Reaction at All-pH. *ACS Sustainable Chem. Eng.* **2019**, *7*, 9, 8993-9001.

[23] Xianwei Lv; Wenwen Tian; Yuping Liu; Zhong-Yong Yuan. Well-defined CoP/Ni_2P nanohybrids encapsulated in a nitrogen-doped carbon matrix as advanced multifunctional electrocatalysts for efficient overall water splitting and zinc–air batteries. *Mater. Chem. Front.* **2019**, *3*, 2428-2436.

[24] Minghao Zhuang; Xuewu Ou; Yubing Dou; Lulu Zhang; Qicheng Zhang; Ruizhe Wu; Yao Ding; Minhua Shao; Zhengtang Luo. Polymer-Embedded Fabrication of Co_2P Nanoparticles Encapsulated in N,P-Doped Graphene for Hydrogen Generation. *Nano Lett.* **2016**, *16*, 7, 4691-4698

[25] Zejun Zhao; Zhixiao Zhu; Xiaobing Bao; Fang Wang; Sijia Li; Shujuan Liu; Yong Yang. Facile Construction of Metal Phosphides (MP, M = Co, Ni, Fe, and Cu) Wrapped in Three-Dimensional N,P-Codoped Carbon Skeleton toward Highly Efficient Hydrogen Evolution Catalysis and Lithium Ion Storage. *ACS Appl. Mater. Interfaces* **2021**, *13*, 9820-9829.
